# Dual cross-attentive mutual teaching for semi-supervised 3D medical segmentation

**DOI:** 10.1371/journal.pone.0352358

**Published:** 2026-06-30

**Authors:** Weiping Ma

**Affiliations:** College of Electronics and Communication Engineering, Lanzhou university of arts and science, Lanzhou, Gansu, China; Jiangsu Open University, CHINA

## Abstract

Semi-supervised learning can reduce the dependence on large-scale labeled data in 3D medical image segmentation.In this work, we propose a new Dual Crossed Attention Mutual Teaching (DCA-MT) framework that effectively utilizes both labeled and unlabeled data by integrating high-dimensional feature alignment, semantic-level crossed attention, and bidirectional knowledge distillation. Specifically, we employ a two-branch VNet architecture where the teacher-student network co-evolves through mutual mentoring and collaborative learning.To enhance representation consistency, we introduce maximum mean difference (MMD) loss and inter-class and intra-class contrast constraints to achieve global feature distribution alignment and class-level separability. A multi-head cross-attention module is designed to facilitate fine-grained semantic interaction between the two networks, allowing the two branches to dynamically exchange complementary features.In addition, the two-way mutual distillation strategy ensures that teacher and student networks benefit from each other's knowledge. Numerous experiments on the left atrial and pancreatic nih datasets show that our proposed approach has better performance and verifies the effectiveness and robustness of DCA-MT.

## 1. Introduction

In the development process of deep learning, semi-supervised learning has gradually become a feasible means to solve high-cost labeling problems [1,2,3,4,5]. Faced with the practical dilemma of constantly expanding data volume and high difficulty of manual annotation, how to improve model performance with massive unannotated data has become a key challenge in the field of computer vision and medical image processing [6]. Medical image segmentation tasks have high requirements for accurate positioning of structural boundaries, but often rely on professional doctors to annotate 3D images manually, which is not only time-consuming and labor-intensive, but also has subjective bias, making high-quality annotation extremely limited, and further amplifies the important value of semi-supervised learning. Existing semi-supervised learning methods generally use a small number of labeled samples as supervised signals and combine unlabeled data for collaborative training to enhance the generalization ability of models in real tasks. Common strategies include consistency regularization [7,8] and pseudo label generation [8,9]. By means of data enhancement, the consistency method requires the model to maintain prediction stability under different disturbance conditions [10]. The pseudo-labeling method uses the output of the current model as a “soft supervision” to guide the model to learn on unlabeled data. For example, Mean Teacher (MT) method [9] uses exponential moving average (EMA) to generate a stable teacher model and introduces consistency loss to improve the robustness of student network. Noisy Student [11] further introduces noise disturbance into the student model, thereby enhancing the tolerance and expression ability of the model for false labels. Although these methods improve the quality of pseudo-labels and the robustness of the model to a certain extent, they are prone to problems such as the accumulation of pseudo-label noise and the slow updating of the teacher's model in the long-cycle training, resulting in limited model generalization ability. In order to improve the feature utilization efficiency of unlabeled data, recent studies have proposed the unified integration of consistent regularization and pseudo-supervision mechanism to build a more stable collaborative training framework [12,13,14]. For example, the MCF method [13] uses heterogeneous structures to learn in parallel, maintaining diversity while generating pseudo-labels. However, such methods are often difficult to ensure the semantic alignment and feature expression collaboration between teachers and students at the same time, especially in the feature space consistency and cross-model interaction.

To solve these problems, this paper proposes a semi-supervised 3D image segmentation framework based on high-dimensional feature alignment and dual-model mutual learning. By constructing a collaborative architecture of two VNet branches, the method integrates high-dimensional distributed alignment (MMD), semantic feature convergence and dispersion loss, cross-attention module and bidirectional distillation mechanism, and significantly enhances the model's ability to express unlabeled data and pseudo-label stability. Specifically, we first construct a two-branch teacher-student structure based on VNet, and update the teacher network with an exponential sliding average strategy to provide stable output. Then, the high dimensional feature distribution alignment loss (MMD) is introduced to align the high dimensional representation of teacher and student in the top layer of the encoder to narrow the feature distribution gap. At the same time, we propose intra-class compression and inter-class stretch constraints to further enhance semantic separability, so that the model can form a clearer foreground/background boundary in the high-dimensional feature space.

To address the above limitations, we propose Dual Cross-Attentive Mutual Teaching (DCA-MT), a semi-supervised framework tailored for 3D medical segmentation, where the supervision is sparse and the anatomical boundaries are sensitive to feature inconsistency. Different from many existing dual-teacher or mutual-learning paradigms that primarily rely on prediction-level consistency or one-way pseudo-supervision, DCA-MT explicitly targets a key conceptual gap: the lack of fine-grained, semantic-level interaction and feature-space coordination between co-evolving networks, which often leads to feature misalignment and unstable pseudo labels when training with large-scale unlabeled volumes. Concretely, we build two parallel VNet branches and introduce a bidirectional cross-attention module at the high-level semantic representation stage, enabling each branch to query the other branch’s deep features and absorb complementary structural cues before decoding. This design forms an explicit semantic exchange pathway beyond rough feature fusion or output imitation, and thus facilitates more reliable mutual correction in challenging 3D scenarios.

Moreover, we design a bidirectional mutual distillation strategy to allow knowledge to flow in both directions: the student benefits from the teacher’s stable supervision derived from labeled data, while the teacher is also encouraged to adapt by absorbing the student’s newly captured structural patterns from unlabeled data. This two-way learning scheme mitigates the common issue where an EMA teacher may update slowly and become less responsive to the evolving distribution of unlabeled samples. To further stabilize collaborative learning in the latent space, we incorporate high-dimensional feature alignment using MMD and introduce inter-class / intra-class semantic constraints to enhance category separability and reduce foreground–background confusion. With these components jointly optimized, DCA-MT enables the two branches to co-evolve consistently at both the feature level and the prediction level, leading to improved segmentation accuracy and generalization under limited annotations.

The main contributions are summarized as follows:

(1) We propose a semi-supervised 3D medical image segmentation framework, DCA-MT, which couples feature-space alignment with mutual teaching to better exploit unlabeled volumes under low-label settings.
(2) We introduce a high-dimensional alignment objective consisting of MMD-based distribution matching and inter-/intra-class constraints, which jointly improve latent-space consistency and semantic separability.
(3) We develop a cross-branch bidirectional cross-attention module that enables semantic-level feature exchange between teacher and student branches, providing a structured interaction pathway beyond prediction-level consistency.
(4) We design a bidirectional mutual distillation mechanism that allows the teacher to remain adaptive to unlabeled data while maintaining stable supervision, thereby improving collaborative learning robustness.

Extensive experiments on two benchmark datasets, Left Atrium and Pancreas-NIH, demonstrate that DCA-MT consistently outperforms competitive semi-supervised baselines, particularly in boundary-sensitive metrics, validating both the effectiveness and robustness of the proposed framework.

## 2. Related work

### 2.1. Consistency regularization

Consistency regularization is a fundamental principle in semi-supervised learning, aiming to enforce prediction invariance under different perturbations or augmentations of the same input, thereby improving robustness and generalization 9,10. Representative approaches include the Π-model 9, which applies stochastic perturbations during training, and Mean Teacher (MT) 19, which stabilizes targets via an exponential moving average (EMA) teacher. Building upon these foundations, many medical segmentation methods further incorporate structural priors or task-level consistency. For example, SASSNet 11exploits anatomical shape constraints, while DTC 12enforces dual-task consistency to enhance representation stability. CPCL 29introduces cyclic prototype consistency, and recent works also explore self-/cross-image consistency to improve robustness across scenes or domains [15] 36. Different from prior methods that mainly enforce consistency at the prediction level, our framework emphasizes cross-network consistency simultaneously in feature space (via global distribution alignment) and semantic space (via cross-attentive interaction), enabling richer and more controllable mutual refinement across training iterations.

### 2.2. Pseudo label generation

Another fundamental strategy in semi-supervised learning is pseudo label generation, which enhances model discriminability by generating supervisory signals from confident predictions on unlabeled data [16]. Existing approaches fall into two broad categories: confidence-based selection and structural refinement. In the former, models like [8,17] filter pseudo labels using confidence thresholds; in the latter, more sophisticated strategies aim to reduce noise and improve label fidelity. Notable examples include SsaNet [18], which employs a trust evaluation module to refine pseudo labels, and UA-MT [19], which leverages uncertainty estimation to filter unreliable predictions. Co-BioNet [20] integrates feedback mechanisms across dual networks to assess uncertainty and retain high-confidence outputs. Tri-Net [21], on the other hand, utilizes two subnetworks to generate pseudo labels for a third model. Other methods like [22] incorporate clustering algorithms such as SLIC for spatial refinement. MCF [13] dynamically generates pseudo labels using a heterogeneous network ensemble, while DeSCO [23] enhances spatial coherence via orthogonal slice analysis in 3D medical data.

Our DCA-MT takes a different perspective by leveraging temporal diversity between models trained at different stages. Instead of relying on a single static prediction, our framework employs cross-iteration comparisons and dual mutual learning to extract high-quality pseudo labels. This approach not only improves reliability but also diversifies the pseudo labels, leading to better model generalization and robust performance gains.

### 2.3. Multi-model frameworks

Multi-model frameworks are widely adopted to introduce diversity and complementarity in semi-supervised segmentation. MT [9] is a canonical example that couples a student with an EMA teacher. Beyond MT, CPS [24] performs cross pseudo supervision via co-training, and CPC [25] exploits confidence-weighted collaborative training. Recent methods further improve mutual learning dynamics, such as heterogeneous ensembles (MCF [13]) or alternating teacher updates (Dual Teacher [26]).

However, many mutual-teaching frameworks still rely on prediction-level agreement and treat knowledge transfer largely as one-way or loosely coupled guidance, which may limit semantic interaction and slow down adaptation on unlabeled distributions. Our framework introduces (i) high-dimensional distribution alignment and class-structured constraints, and (ii) a dual cross-attention bridge that enables teacher and student to query each other in the deep semantic space, thereby forming a more explicit and fine-grained mutual refinement loop.

### 2.4. Cross-domain consistency and representation learning

Beyond medical segmentation, recent advances in other vision and signal processing domains have highlighted the importance of structured representation optimization, multi-granularity learning, and generative modeling under limited supervision. For example, time-frequency aware hierarchical feature optimization has been explored for robust recognition under complex interference [27] 37, and multilevel contrastive learning has been shown effective for few-shot recognition with stronger discriminability [28] 38. In multimodal semantic segmentation, deformation-resilient multigranularity learning is proposed to handle modality misalignment and structural deformation [29] 39, suggesting that hierarchical constraints can improve robustness under challenging conditions. Generative modeling also provides complementary perspectives. Diffusion-based methods that explicitly model distribution differences have been proposed to improve recognition and calibration under unknown or shifting distributions [30] 40. Moreover, domain-attentive contrastive learning has been investigated to enhance cross-domain feature extraction from unlabeled data, demonstrating the effectiveness of structured contrastive objectives [31]41. Meanwhile, self-/cross-image consistency learning has been studied for remote sensing segmentation, further supporting the value of explicit cross-sample constraints [32] 36.

Motivated by these insights, our DCA-MT combines distribution alignment, class-structured feature constraints, and explicit cross-network semantic interaction to build a closed-loop mutual teaching framework that better exploits unlabeled data in 3D medical segmentation.

## 3. Overall architecture of DCA-MT

**Fig 1:**
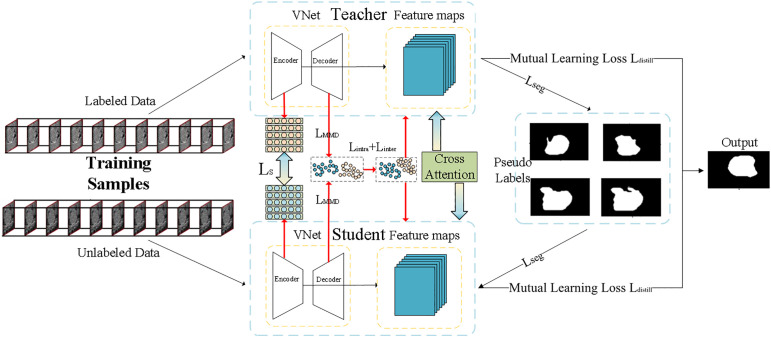
Architecture of DCA-MT.

An overview of the proposed DCA-MT (Dual Cross-Attentive Mutual Teaching) framework is presented in Fig 1. The architecture is composed of two parallel VNet-based encoder-decoder branches, each representing a Teacher and a Student network, respectively. These two networks interact with each other through a high-level Multi-Head Cross-Attention Module, which is embedded at the deepest encoding layer to facilitate semantic-level feature exchange and alignment.

### 3.1. Dual-branch encoder design

Both branches adopt the standard 3D VNet encoder [33] consisting of five hierarchical convolutional blocks. Each block contains a series of 3 × 3 × 3 convolutional layers followed by ReLU activation functions. The number of feature channels doubles progressively from shallow to deep layers (16 → 32 → 64 → 128 → 256), enhancing the network’s ability to capture multi-scale semantic patterns, which is crucial for segmenting anatomical structures with varying shapes and sizes.

To preserve spatial context, each level employs a stride-2 convolution or MaxPool3d for downsampling, reducing the resolution in stages (256³ → 128³ → 64³ → 32³ → 16³). The resulting deep feature maps are then reshaped into sequences of size B × (8 × 8 × 8) × 256, preparing them for the attention module.

### 3.2. Cross-attention mechanism

To overcome the limitations of conventional self-attention, we introduce a Bidirectional cross-attention module, where the Teacher and Student features serve as both Queries and Keys/Values for each other. This allows for deep feature-level interaction between branches. We adopt an 8-head attention mechanism, where each head projects the input into 32-dimensional subspaces and aggregates the results into a 256-channel output. This design enhances inter-branch information flow and mitigates the inefficiency of unidirectional distillation or naive feature fusion methods.

#### 3.2.1. Decoder and skip connections.

After attention-based interaction, the updated high-level features are routed back to their respective decoders. Each decoder performs progressive upsampling via transposed 3D convolutions (kernel_size = 2, stride = 2), restoring resolution step-by-step from 8³ to 16³, 32³, 64³, and back to full size. Channel dimensions are correspondingly reduced from 256 → 128 → 64 → 32 → 16. At every decoding level, skip connections are utilized to fuse high-resolution encoder features, preserving spatial details critical for accurate boundary delineation. The final output volume is projected to a 2-channel probability map via a 1 × 1 × 1 convolution and a softmax activation.

#### 3.2.2. Multi-level loss design.

To ensure effective learning, the model incorporates a composite loss function:

(1) Supervised Loss: A weighted sum of Dice loss and Cross-Entropy loss is used for labeled data.
(2) Dual-Direction Distillation Loss: The Teacher supervises the Student via output alignment, while the Student also transfers its learned knowledge from unlabeled data back to the Teacher.
(3) Feature Alignment Losses: At the deep feature level, we impose MMD loss and inter-/intra-class contrastive losses to enforce global distribution alignment and enhance category-level feature separability.

All these losses are integrated and jointly optimized during backpropagation, enabling the framework to balance prior knowledge learning from labeled data and structure discovery from unlabeled data. This results in more robust and fine-grained 3D segmentation performance.

### 3.3. High-dimensional feature alignment

Distribution alignment has become a fundamental concept in transfer learning and semi-supervised learning. Maximum Mean Discrepancy (MMD) is a widely used statistical measure for comparing two distributions via their mean embeddings in a Reproducing Kernel Hilbert Space (RKHS). Unlike KL divergence, which operates at the output level, MMD measures the discrepancy in the latent feature space, making it well-suited for aligning high-dimensional representations in semi-supervised settings. Let 𝑃 and 𝑄 denote the empirical feature distributions extracted from the Teacher and Student encoders, respectively. Given a mini-batch of paired inputs, we define the feature representations from the Teacher encoder as: $\phi_{t}=\{{\phi_{t}}^{\left(1\right)},{\phi_{t}}^{\left(2\right)},...,{\phi_{t}}^{\left(n\right)}\},{\phi_{t}}^{\left(i\right)}\in {\mathbb{R}}^{d},$ and from the Student encoder as: $\phi_{s}=\{{\phi_{s}}^{\left(1\right)},{\phi_{s}}^{\left(2\right)},...,{\phi_{s}}^{\left(n\right)}\},{\phi_{s}}^{\left(i\right)}\in {\mathbb{R}}^{d},$ where d is the dimensionality of the feature space. The squared MMD between 𝑃 and 𝑄 is defined as the distance between their kernel mean embeddings:


$$\begin{array}{c} MMD^{\text{2}}\left(P,Q\right)=\overset{\text{2}}{\underset{H}{\|E_{\varphi_{t}\;P}\left[\phi \left(\varphi_{t}\right)\right]-E_{\varphi_{s}\;Q}\left[\phi \left(\varphi_{s}\right)\right]\|}} \end{array}$$ (1)

where, ϕ(·) is the kernel mapping function into the RKHS 𝓗.

#### 3.3.1. MMD in our model.

In practice, we adopt the Radial Basis Function (RBF) kernel to approximate MMD efficienty:


$$\begin{array}{c} MMD^{\text{2}}=\frac{\text{1}}{n^{\text{2}}}k\left({\varphi_{t}}^{\left(i\right)},{\varphi_{t}}^{\left(j\right)}\right)+\frac{\text{1}}{n^{\text{2}}}k\left({\varphi_{s}}^{\left(i\right)},{\varphi_{s}}^{\left(j\right)}\right)-\frac{\text{2}}{n^{\text{2}}}k\left({\varphi_{t}}^{\left(i\right)},{\varphi_{s}}^{\left(j\right)}\right) \end{array}$$ (2)

where the RBF kernel is defined as:


$$\begin{array}{c} K\left(x,y\right)=exp(-\frac{{\|x-y\|}^{\text{2}}}{2\sigma^{\text{2}}}) \end{array}$$ (3)

and σ is the kernel bandwidth. A larger σ leads to smoother similarity estimation, while a smaller σ increases sensitivity but may cause overfitting. By minimizing the MMD loss between the Teacher and Student feature distributions, we encourage global feature alignment, reduce representational mismatches, and promote stable collaborative learning between the two branches.

#### 3.3.2. Inter and intra-class feature losses.

Distinguishing the target organ or lesion (foreground) from other tissues (background) is a core task. Although MMD can realize the alignment of the overall feature distribution of teachers and students, it does not explicitly distinguish the convergence and dispersion of different semantic categories (such as foreground and background) in the feature space. This means that even if the overall distribution of Teacher and Student is aligned, there may be a problem of confusion between foreground and background characteristics. Therefore, Inter-Class/Intra-Class Feature Loss is introduced in this chapter to further constrain the feature compactness of similar samples and the feature separability of heterogeneous samples. Suppose there are N samples in a batch; For “unlabeled” samples, we approximate labeling using predictions (false labels) given by the teacher model. For the i th sample, its 3D label is, where 1 represents the foreground; In many cases, we can use global pooling or other methods to get a certain foreground activation rate, for example, if the sample contains the foreground as a whole, it is regarded as “foreground sample”; In addition, we want features of the same category (foreground or background) to be close to each other in a high-dimensional space, reflecting “homogeneity compact.” A simple way to write it is to calculate and average the characteristic distance between teacher and student for all sample pairs that satisfy “generic foreground” or “generic background”:


$$\begin{array}{c} L_{intra}=\frac{\text{1}}{\left|P_{same}\right|}\sum_{\left(i,j\right)\in P_{same}}({\|t_{i}-t_{j}\|}_{\text{2}}+{\|s_{i}-s_{j}\|}_{\text{2}}) \end{array}$$ (4)

where,$P_{same}$ represents a collection of pairs of samples of the same class. If we consider the foreground and background separately, we can also add the foreground and background pairs separately. This loss encourages the feature spacing of homogeneous samples to become smaller, resulting in a more compact distribution in the feature space.

Compared with $L_{intra}$, we hope that the feature spacing of different classes should be as large as possible to make the decision boundary clearer. We define as $L_{inter}$:


$$\begin{array}{c} L_{inter}=\frac{\text{1}}{\left|P_{diff}\right|}\sum_{\left(i,j\right)\in P_{diff}}({\|t_{i}-t_{j}\|}_{\text{2}}+{\|s_{i}-s_{j}\|}_{\text{2}}) \end{array}$$ (5)

where,$P_{diff}$ represents pairs of samples of different classes. The reason for the minus sign is that in optimization we minimize $L_{inter}$ and want the distance between different classes to be as large as possible, that is, minimizing the negative distance is equivalent to maximizing the distance.

#### 3.3.3. Combined feature alignment objective.

In the case of labels, if we have real labels for each sample, we can accurately distinguish which voxels belong to the foreground or background, so as to determine the relationship between $P_{same}$ and $P_{diff}$. In the absence of labels, the prediction of the teacher model on the unlabeled data or the combination of teacher/student predictions can be used to generate false labels; Pseudo-labels are used to approximate the classification. Although the pseudo-label may have noise, it can still effectively improve the discriminating power of the feature distribution under the average action of a large number of unlabeled data. The role of MMD loss in the model is to align the global teacher-student distribution so that it has smaller overall differences in the same feature space. The $L_{intra}$/$L_{inter}$ can further distinguish the distribution structure of different semantic classes in this alignment space to ensure the compactness within the same class and the separability between different classes. So the two actors complement each other. If only MMD loss is used, the problem of feature alignment but foreground and background confusion may occur. If only $L_{intra}$/$L_{inter}$ are used, the global statistics for teachers and students may be difficult to align. So in our framework, $L_{inter}$,$L_{intra}$, and $L_{MMD}$ appear together in weighted form, as:


$$\begin{array}{c} L_{S}=\alpha L_{MMD}+\beta L_{intra}+\gamma L_{inter} \end{array}$$ (6)

### 3.4. Cross-attention for mutual enhancement

In the double-branch Teacher-Student scenario concerned by this research, if only self-attention within a single network is used, it is difficult to solve the problem of insufficient cross-network information interaction. Therefore, we construct a cross-attention module: perform the “S → T” and “T → S” attention operations respectively between the high-level features of the teacher-student branch, so that the Teacher can query based on the features of the Student and integrate the useful information into the output of the Teacher. And vice versa. This two-way information interaction breaks the limitation of traditional one-way distillation or simple feature stitching, and can improve the feature fusion effect under the combination of sparse and large-scale unlabeled data. For visual tasks, if the image or voxel features are mapped into a sequence form (for example, N represents the number of spatial locations and d represents the number of channels), the self-attention can capture the dependence between any two places in the same image (or the same feature mapping), so as to learn more global and local associations. However, in the two-branch medical image segmentation architecture proposed in this chapter, relying only on self-attention within a single network is still insufficient to fully explore the “cross-network” information communication. In addition, in the multi-branch network structure, there are usually such cases: the first branch: obtains better foreground discrimination ability on labeled data; The second branch: Learn broader background deformation patterns and underlying structures with the help of large scale unlabeled data. If only one-way distillation is relied on, information transmission is likely to be superficial: students passively receive the teacher's prior, and the teacher can not absorb the students’ understanding of the new data distribution; And the traditional feature fusion is often just the simple splicing or addition of features, lack of pertinence or lack of attention mechanism fusion.

**Fig 2:**
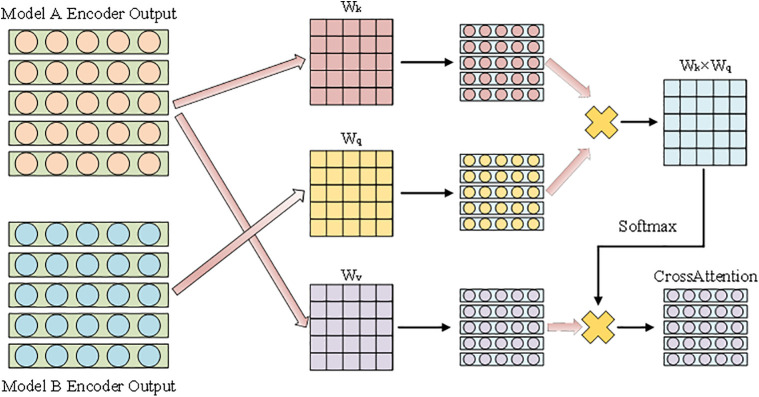
Structure of cross-attention mechanism.

To this end, the cross attention module is introduced, as shown in Fig 2. First, it enables students to obtain more discriminative prospect information by querying teacher characteristics. Second, let the teacher's characteristics absorb the background diversity and noise patterns mastered by the students in the data by querying the student characteristics. This two-way information interaction effectively breaks through the limitations of traditional distillation or splicing, making both branches dynamically updated in high-dimensional space. Specifically, the teacher and student models output the highest-level encoder feature maps respectively, and first represent their reshape sequences with length and channels (i.e.,). The input features are then linearly mapped to generate Query, Key, and Value vectors, respectively. In the Attention calculation, students’ features are used as Query and teachers’ features as Key and Value, which constitute the path of “students inquiring teachers.” And vice versa. The attention feature of the final output is the cross-model semantic enhancement representation, feedback back to the original model path and participate in the subsequent decoding process. In terms of implementation, the cross-attention module adopts a multi-head mechanism, setting the number of heads to 4 and each head dimension to 32, to ensure that the model can simultaneously capture fine-grained association information of multi-scales, different structures or anatomical regions. Different from traditional self-attention, this module is not used to model the internal features of the same model, but to build an “external connection path” between the two models, which acts as a bridge and strengthens the semantic consistency of mutual learning. It is worth emphasizing that this module not only improves the efficiency of feature alignment between the two models, but also provides a high-quality intermediate semantic basis for the mutual learning mechanism, making the bidirectional distillation more stable and reliable. On the whole, the cross-attention mechanism not only serves to optimize the model structure, but also serves as the intermediate link of the dual-model collaborative updating mechanism.

### 3.5. Bidirectional mutual distillation

Knowledge distillation generally assumes that the teacher model performs better on a particular dataset or task, and the student model has less model size or computational overhead, and thus needs to improve accuracy by mimicating the teacher's predictive distribution or intermediate features. However, in the semi-supervised scene of 3D medical imaging, the student model is not only learning on the same data set as the teacher, but is also exposed to a large number of unlabeled data, from which new structural patterns and deformation experiences may be gained. If the teacher model always stays on the only labeled data and cannot dynamically update its cognition of the unlabeled data, the teacher's guidance to students may appear “outdated” or “incomplete information” phenomenon. In some cases, teachers may have biased or even false priors on unlabeled data, limiting overall performance gains. The distillation goal is usually to have students mimic the teacher's output (such as logits) or feature distribution (such as middle layer features), resulting in one-way learning:


$$\begin{array}{c} L_{distill}\left(S\right)=D_{KL}\left(\sigma \left(z_{T}/T_{temp}\right),\sigma \left(z_{S}/T_{temp}\right)\right) \end{array}$$ (7)

where, $z_{T}$ and $z_{S}$ are logits for teachers and students respectively, σ is softmax function and $T_{temp}$ is temperature coefficient. However, in the semi-supervised scenario, the “student” is not simply trained on the same data set, but is exposed to a large amount of additional unlabeled data. If students learn new feature patterns or background contexts from these unlabeled data, but cannot return to the teacher model for updating, then the teacher model remains on the limited prior with labeled data. As a result, the segmentation effect of the teacher model on unlabeled data may not be improved, and even misguidance may occur in some extreme cases.

Based on the above considerations, we design a Two-Way mutual learning mechanism, so that in a training iteration, both teachers guide students and students feed teachers, and the traditional distillation loss of teacher guide students is as follows:


$$\begin{array}{c} L_{TS}=D_{KL}\left(\sigma \left(z_{T}\right),\sigma \left(z_{S}\right)\right) \end{array}$$ (8)

It means that the student should be as close as possible to the teacher's predicted probability distribution or feature representation. By combining MMD with inter-class/intra-class losses mentioned above, the distribution of students in the middle tier can be brought closer to the teacher. The reverse distillation losses reported by students to teachers are:


$$\begin{array}{c} L_{ST}=D_{KL}\left(\sigma \left(z_{S}\right),\sigma \left(z_{T}\right)\right) \end{array}$$ (9)

It means that teachers should also absorb the predicted distribution of students to a certain extent to adapt to the scenario where the unlabeled data is richer. Cross-attention fusion: Teachers can query student features during high-level feature calculation to obtain knowledge of background context or variable anatomy.

Finally, the distillation losses in these two directions can be written as:


$$\begin{array}{c} L_{M}=\lambda_{\text{1}}L_{TS}+\lambda_{\text{2}}L_{ST} \end{array}$$ (10)

where, $\lambda_{\text{1}}$ and $\lambda_{\text{2}}$ are the equilibrium coefficients. In the actual network training, after a batch is input, the teacher and the student do forward calculation respectively to obtain the feature T,S and output logits $z_{T}$,$z_{S}$. After the cross-attention mechanism, T and S are updated to T ' and S ‘. Calculate the separation loss $L_{seg}$, alignment loss $\alpha L_{MMD}+\beta L_{intra}+\gamma L_{inter}$, and bidirectional distillation loss $L_{distill}$; After these losses are accumulated, they are backpropagated, and the model parameters of teacher and student are updated to achieve the effect of two-way learning.

## 4. Experiments

### 4.1. Implementation settings

Fair Comparison Protocol. To ensure a fair comparison, we implement all competing semi-supervised segmentation methods using the same 3D segmentation backbone unless explicitly stated otherwise. Specifically, VNet is adopted as the default backbone for our method and all baseline methods (UA-MT, SASSNet, DTC, BCP, MCF, and GA). All methods share the same preprocessing pipeline, data splits, patch sampling strategy, and sliding-window inference setting on each dataset. For baselines that provide official implementations or method-specific schedules, we follow the original training protocol as closely as possible and report any deviations or required adaptations to the VNet backbone for transparency. For EVIL-3D [34], the original method was designed for 2D segmentation (ACDC, MM-WHS, MonuSeg) using a 2D U-Net backbone. To enable fair comparison on our 3D benchmarks, we re-implement EVIL as a 3D adaptation: the E-Net branch is built upon VNet with an additional evidential output head (Dirichlet parameterization via softplus activation), while the S-Net branch uses a standard VNet. The uncertainty threshold T = 0.2 for pseudo-label filtering follows the original paper. Both branches are trained with the same supervised Dice+CE loss on labeled data, and S-Net receives uncertainty-masked pseudo labels from E-Net on unlabeled data, consistent with the original design. All other training settings (optimizer, learning rate schedule, patch size, batch size) are identical to those used for the other baselines.

Optimization and Training Strategy. All experiments are conducted under the same semi-supervised setting. Each mini-batch contains both labeled and unlabeled samples (batch size 4, with 2 labeled + 2 unlabeled). We train the networks for 6000 iterations using SGD with momentum 0.9 and weight decay 1e − 4. The initial learning rate is set to 0.01 and decayed by a factor of 10 after every 2500 iterations. For teacher updates, we use an EMA strategy with decay rate 0.99/0.999. For data augmentation, we apply standard 3D spatial transforms and intensity perturbations following prior semi-supervised segmentation works. At inference, we adopt a sliding-window strategy with dataset-specific patch sizes and strides (LA: 112 × 112 × 80, stride 18 × 18 × 4; Pancreas: 96 × 96 × 96, stride 16 × 16 × 16) to obtain full-volume predictions.

Loss Weights and Hyperparameter Selection. The overall objective follows Eq. (6) and Eq. (10). The balancing coefficients are set as α = 1.0, β = 0.5, γ = 0.1 for feature alignment/separability losses and λ₁ = 1.0, λ₂ = 0.3 for bidirectional distillation. These values were initialized based on common practices in related works and further tuned using a held-out validation subset via grid search, selecting the configuration that yields the best Dice and 95HD.

All experiments are conducted on a workstation equipped with an NVIDIA GeForce RTX 4090 GPU under Ubuntu 20.04 using PyTorch 2.4.0. We fix the random seed to 42 to reduce variance.

### 4.2. Datasets and evaluation metrics

To evaluate the effectiveness and generalization capability of our approach, we conduct experiments on two distinct 3D medical imaging datasets, each representing a different modality. We adopt a consistent 80/20 split for training and testing purposes, and the portion of labeled samples is selected from the training subset only.

#### 4.2.1. LA dataset.

The Left Atrial dataset [35] comprises 100 high-resolution 3D MR volumes enhanced by gadolinium contrast agents, each with an isotropic voxel size of 0.625 mm³. Ground truth annotations delineating the left atrium are provided. In the data preparation pipeline, we first normalize each volume to have zero mean and unit variance. Next, volumes are cropped to center around the region of interest with extended boundaries to ensure full coverage. During training, random sub-volumes of size 112 × 112 × 80 are extracted as input patches. At inference, a sliding window strategy with strides of 18 × 18 × 4 is employed to generate the full segmentation output.

#### 4.2.2. Pancreas-NIH dataset.

This dataset [36] contains 82 contrast-enhanced abdominal CT scans, each accompanied by pixel-wise annotations for the pancreas. The CT volumes vary in depth, with shapes of 512 × 512 × D where D ∈ [181, 466]. We apply a soft-tissue windowing of [−120, 240] HU to standardize intensity values. Cropping is performed around the pancreas center with an additional 25-voxel padding. Training inputs are obtained by randomly cropping patches of 96 × 96 × 96 voxels. For evaluation, we adopt a sliding inference approach with the same window size and strides of 16 × 16 × 16.

#### 4.2.3. Evaluation metrics.

To comprehensively assess segmentation performance, we adopt four quantitative metrics commonly used in prior studies [37,38,39,13,40,19]. These include region-based measures—Dice Similarity Coefficient (Dice) [19] and Jaccard Index [39]—as well as boundary-sensitive metrics—95th Percentile Hausdorff Distance (95HD) [40] and Average Surface Distance (ASD) [37].

### 4.3. Ablation study

To evaluate the individual contribution of each proposed component, we conducted a comprehensive ablation study on both the Pancreas-NIH and LA datasets. In each experiment, only the component under investigation was altered, while all other settings remained fixed for a fair comparison.

The full version of our method, denoted as All, incorporates four major components: (1) high-dimensional feature alignment via MMD, (2)$L_{intra}$/$L_{inter}$, (3) the Cross-Attention Module for feature exchange between Teacher and Student, and (4) the Mutual Distillation Mechanism. To better understand the role of each module, we successively remove them from the full model, forming the following baselines:

(1) Base: A dual-branch VNet model without any of the proposed modules, serving as the backbone.
(2) Loss: Incorporates only the MMD-based distribution alignment and semantic contrastive losses.
(3) CrossAttention: Adds the cross-attention module to the base model.
(4) Mutual: Adds the Bidirectional mutual distillation mechanism without attention or feature alignment.

The quantitative results are summarized in Table 1 and Table 2. As shown, the base model exhibits limited segmentation capability under sparse supervision, especially on boundary-sensitive metrics such as 95HD and ASD. Adding only the feature alignment loss (Loss) significantly improves Dice and Jaccard, indicating the effectiveness of high-dimensional supervision.The CrossAttention model outperforms the base by a notable margin, demonstrating that interactive attention between Teacher and Student branches provides valuable semantic enhancement. Introducing the Mutual learning strategy further improves overall consistency, particularly reducing the surface-based errors. Finally, the full All model—integrating all proposed components—achieves the best performance across all metrics. These results validate the complementary nature of each design module and demonstrate the robustness of the proposed DCA-MT framework in learning from limited annotations.

**Table 1:** Ablation experiments on the LA dataset.

Model Name	Dice	Jaccard	95HD	ASD
All	83.65	72.59	3.21	1.37
Base	74.40	59.94	6.32	1.77
Loss	81.30	68.63	3.54	1.97
CrossAttention	77.48	63.54	4.88	1.41
Mutual	82.98	71.44	3.56	1.42

**Table 2:** Ablation experiments on the Pancreas dataset.

Model Name	Dice	Jaccard	95HD	ASD
All	93.74	88.32	2.54	1.42
Base	77.29	64.58	10.89	3.39
Loss	88.26	79.13	5.53	1.67
CrossAttention	89.57	81.18	4.80	1.43
Mutual	90.47	82.73	4.65	1.68

**Fig 3:**
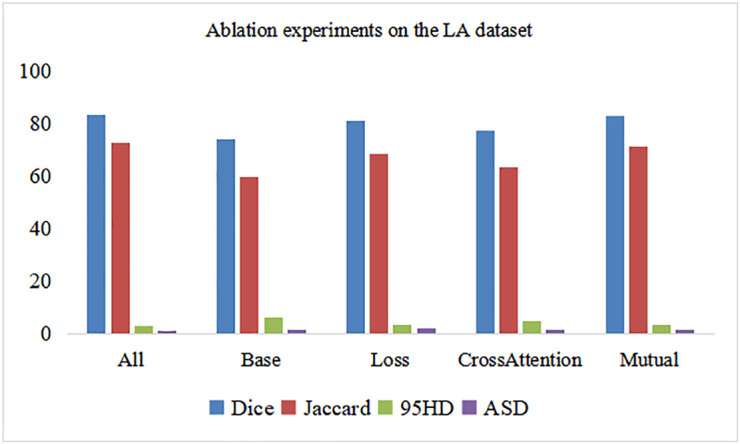
Performance on the LA dataset.

**Fig 4:**
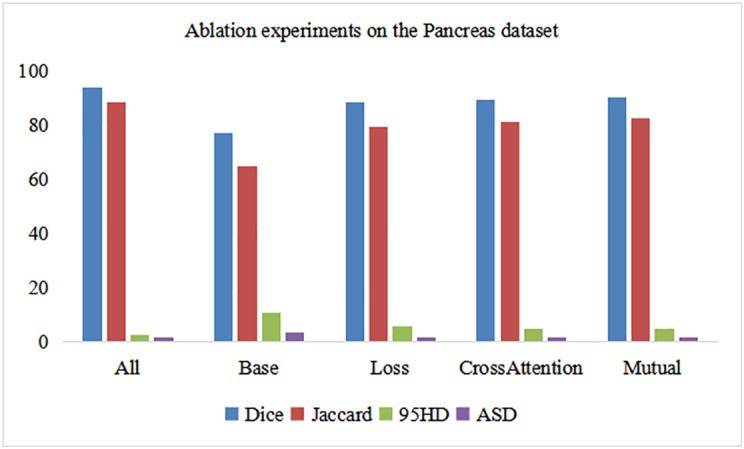
Performance on the Pancreas dataset.

To evaluate the individual effectiveness of each proposed component, we performed comprehensive ablation studies on both the LA and Pancreas-NIH datasets, as shown in Fig 3 and Fig 4. The base dual-VNet model, without any enhancements, showed limited segmentation capability, especially in boundary-sensitive metrics, with Dice scores of 74.40% and 77.29% and 95HD values of 6.32 and 10.89 on the LA and Pancreas datasets, respectively. Introducing the high-dimensional feature alignment and semantic contrastive loss (Loss) led to significant performance gains, increasing Dice to 81.30% and 88.26%, and markedly reducing 95HD, which demonstrates the effectiveness of global feature distribution alignment and class separability. The cross-attention module further improved spatial precision and semantic representation, reflected by improvements in Dice and ASD. Meanwhile, incorporating the mutual distillation mechanism yielded strong results across all metrics, highlighting its role in facilitating dynamic knowledge exchange between the teacher and student networks. When all modules were combined, the full DCA-MT model achieved the best performance on both datasets, with Dice scores of 83.65% (LA) and 93.74% (Pancreas), and 95HD values reduced to 3.21 and 2.54, respectively. These results confirm that each module contributes complementary benefits and that their integration leads to robust and accurate segmentation under semi-supervised conditions.

### 4.4. Comparison with other methods

**Fig 5:**
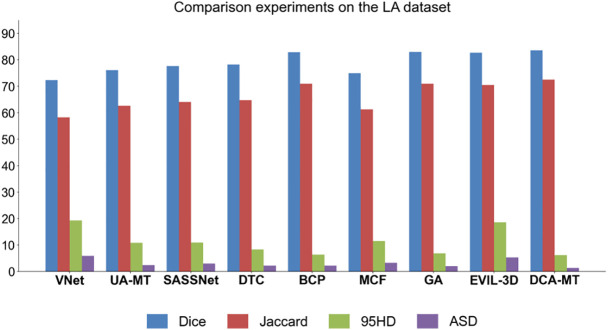
Comparison results on the LA dataset.

To assess the competitiveness of our method, we carried out comparative evaluations against several leading semi-supervised segmentation models using both the LA and Pancreas-NIH datasets. We selected VNet as the foundational benchmark architecture. Among the comparative approaches, we included UA-MT [19], which incorporates uncertainty-aware learning strategies, SASSNet [38], which exploits anatomical shape priors, DTC [39], known for enforcing consistency at the task level, BCP [37], a method based on bidirectional CutMix augmentation [41], and MCF [13], which leverages heterogeneous network ensembles for model-level consistency. We also compared against the recent Gradient-Aware method proposed by Qi et al. [42], which introduces gradient-guided optimization to address class imbalance in semi-supervised segmentation. Both BCP and MCF represent top-performing solutions in current literature. Specifically, for BCP, we adhered strictly to the training schedule detailed in the original paper, involving 2,000 iterations of pre-training followed by 15,000 iterations of self-training. As part of our analysis, we conducted extensive cross-model experiments on the LA dataset, using training subsets containing 10% labeled samples. The corresponding results are presented in Table 3 and Fig 5.

**Table 3:** Comparison experiments on the LA dataset.

Model Name	Dice	Jaccard	95HD	ASD
VNet	72.38	58.26	19.35	5.89
UA-MT	76.10	62.62	10.84	2.43
SASSNet	77.66	64.08	10.93	3.05
DTC	78.27	64.75	8.36	2.25
BCP	82.91	70.97	6.43	2.25
MCF	75.00	61.27	11.59	3.27
GA	83.02	71.04	6.89	2.02
EVIL-3D	82.76	70.53	18.62	5.34
DCA-MT	83.65	72.59	6.21	1.37

From the comparison, our proposed DCA-MT framework outperforms all baseline methods across all evaluation metrics. Specifically, DCA-MT achieves a Dice score of 83.65%, surpassing the strongest baseline BCP (82.91%), while also yielding a substantially lower 95HD (3.21 vs. 6.43) and ASD (1.37 vs. 2.25), indicating more precise boundary localization.Furthermore, compared to the Gradient-Aware method (GA), which also achieves strong Dice performance (83.02%) under class imbalance settings, DCA-MT still leads in both Jaccard (72.59% vs. 71.04%) and boundary-aware metrics such as 95HD (6.21 vs. 6.89) and ASD (1.37 vs. 2.02), demonstrating more robust segmentation consistency at fine-grained spatial levels.

**Fig 6:**
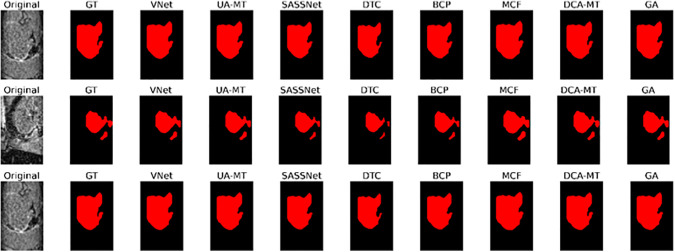
Part of visualization results.

While methods like UA-MT, SASSNet, and DTC demonstrate consistent improvements over the VNet baseline, they still fall short in capturing boundary-level accuracy and semantic structure when compared to our model. The performance gap is especially noticeable in boundary-sensitive metrics such as 95HD and ASD, where DCA-MT achieves >50% reduction in error compared to VNet, and ~50% reduction compared to the best competing method. The superior performance of DCA-MT can be attributed to the synergy of its key components: high-dimensional feature alignment enhances distribution-level consistency; dual cross-attention modules enable rich inter-branch feature exchange; and mutual distillation allows dynamic knowledge adaptation from both labeled and unlabeled data. In addition to numerical results, Fig 6 provides visual comparisons highlighting DCA-MT’s ability to produce cleaner, more complete segmentation maps with sharper organ boundaries and fewer false positives.

### 4.5. Computational cost and efficiency

DCA-MT introduces a dual-branch teacher–student framework with a bidirectional cross-attention module and auxiliary feature-alignment losses, which naturally incurs higher cost than a single-branch backbone as shown in Table 4. All measurements reported here are conducted on an NVIDIA GeForce RTX 3090 (PyTorch 2.4.0, FP32) under the same experimental configuration (VNet backbone, batch size 4 with 2 labeled + 2 unlabeled samples). The single-branch VNet backbone has 9.44M parameters and requires 40.27 GMACs per patch, with a training throughput of 58.8 ms/iteration. DCA-MT maintains two VNet branches (18.88M parameters combined) plus a lightweight bidirectional cross-attention module at the deepest encoder stage, adding only 0.27M parameters—less than 1.5% of the total 19.15M parameter count. Since the cross-attention operates exclusively at the lowest spatial resolution, its computational overhead is negligible: the total GMACs of DCA-MT (80.55 GMACs/patch) is dominated by the two convolutional branches and is on par with MCF (80.55 GMACs/patch), the only other dual-network baseline. In terms of training time, DCA-MT runs at 177.2 ms/iteration, approximately 33% higher than mean-teacher family methods (UA-MT, SASSNet, DTC, BCP, GA: ~ 133 ms/iteration), but 34% lower than MCF (267.2 ms/iteration), whose mutual consistency losses are computationally heavier. During inference, DCA-MT averages the probability maps of both branches without iterative refinement, resulting in an inference time of 4.55 s/volume on LA and 0.66 s/volume on Pancreas—comparable to MCF (4.49 s/vol, 0.69 s/vol) and approximately 1.7 × that of single-branch methods (2.74 s/vol, 0.35 s/vol). This modest overhead is justified by substantially improved segmentation accuracy, particularly on boundary-sensitive metrics: DCA-MT achieves a 95HD of 3.21 mm on LA, which is 50% lower than BCP (6.43 mm) and 53% lower than MCF (11.59 mm) under the same dual-branch budget.

**Table 4:** Computational cost on comparison methods.

Method	Params(M)	GMACs/patch	Train(ms/iter)	Infer-LA(s/vol)	Infer-PA(s/vol)
VNet(backbone)	9.44	40.27	58.8	2.63	0.31
UA-MT	9.44↑	40.27↑	132.6	2.74↑	0.35↑
SASSNet	9.44↑	40.27↑	132.9	2.74↑	0.35↑
DTC	9.44↑	40.27↑	133.1	2.74↑	0.35↑
BCP	9.44↑	40.27↑	134.1	2.74↑	0.35↑
MCF	18.89	80.55	267.2	4.49	0.69
GA	9.44↑	40.27↑	134.0	2.74↑	0.35↑
DCA-MT(ours)	19.15	80.55	177.2	4.55	0.66

## 5. Backbone generalization

Although DCA-MT is instantiated with VNet in our main experiments, the proposed framework is not tied to any specific segmentation backbone. This is because DCA-MT operates on two generic interfaces that are available in most 3D encoder–decoder architectures: (i) a deep semantic feature tensor extracted from the deepest encoder stage, and (ii) the corresponding prediction logits (or probability maps) used for pseudo supervision and distillation. Specifically, the proposed components—including high-dimensional distribution alignment (MMD), inter-/intra-class separability constraints, bidirectional cross-attention, and bidirectional mutual distillation—do not rely on VNet-specific operators (e.g., particular residual paths or skip-connection layouts). The only practical requirement for integrating DCA-MT into another backbone is that the two branches expose deep features with matched spatial resolution; if channel dimensions differ across backbones, a lightweight 1 × 1 × 1 projection layer (or linear mapping after flattening) can be used to align the feature dimension prior to cross-attention computation. Moreover, the cross-attention module is placed at the deepest feature level where the spatial size is smallest, which keeps the additional computational overhead bounded and makes the integration straightforward for alternative networks.

To empirically verify this backbone-agnostic property, we additionally implement DCA-MT on an alternative 3D segmentation backbone, 【Backbone-B: e.g., 3D U-Net / nnU-Net】, under the same semi-supervised protocol and labeled ratio as the VNet setting. As summarized in Table 【X】, DCA-MT consistently improves over the corresponding backbone baseline on 【Dataset: LA / Pancreas】, yielding notable gains in Dice and boundary-sensitive metrics (95HD/ASD). Importantly, the improvement trend remains stable across different architectural designs, indicating that the proposed mutual teaching with semantic-level cross-attention and feature-space alignment provides architecture-independent benefits rather than being over-specialized to VNet. These results support the claim that DCA-MT can serve as a generally applicable training framework for semi-supervised 3D medical segmentation, and it can be extended to other modern backbones (e.g., transformer-based volumetric models) with minor engineering adaptations.

## 6. Conclusion

In this paper, we proposed DCA-MT (Dual Cross-Attentive Mutual Teaching), a novel semi-supervised framework for 3D medical image segmentation. Built upon a dual-VNet architecture, DCA-MT integrates high-dimensional feature alignment, semantic-level cross-attention, and a bidirectional mutual distillation mechanism to fully exploit both labeled and unlabeled data. By aligning the global feature distributions via MMD and enhancing category separability through $L_{intra}$and $L_{inter}$, our model mitigates feature confusion in the latent space. The introduction of a dual cross-attention module enables fine-grained semantic interaction between teacher and student models, while the bidirectional distillation strategy allows both networks to co-evolve and adapt dynamically, alleviating the limitations of traditional one-way supervision.Comprehensive experiments on two benchmark datasets—Pancreas-NIH and LA—demonstrate that DCA-MT consistently outperforms existing state-of-the-art semi-supervised segmentation methods, especially in terms of boundary-aware metrics such as 95HD and ASD. The ablation studies further validate the complementary contributions of each proposed module, and the cross-model comparisons highlight the superiority of DCA-MT in leveraging limited supervision for precise anatomical segmentation. Despite these encouraging results, several limitations should be acknowledged. First, as DCA-MT relies on pseudo supervision and mutual distillation, its performance may degrade when pseudo-label quality is severely affected by strong domain shifts, heavy artifacts, or extremely low labeled ratios, potentially leading to error accumulation. Second, the effectiveness of mutual teaching partly depends on maintaining useful discrepancy between branches; if the two branches become overly similar, the marginal gain from cross-attention and bidirectional distillation may diminish. In addition, early-stage feature exchange may introduce noisy semantics when the model is not yet stabilized, and segmentation of very small structures or highly ambiguous boundaries may still remain challenging. From a scalability perspective, the dual-branch design increases memory and computation, which may be a constraint for large-volume inference or resource-limited deployment. These issues motivate future exploration of more robust pseudo-label filtering (e.g., uncertainty-aware selection), warm-up or schedule-controlled activation of cross-attention/distillation, and parameter-efficient variants that distill the dual-branch knowledge into a lightweight model for deployment. As future work, we plan to explore extending DCA-MT to more practical deployment settings where centralized training is restricted. A particularly promising direction is distributed and federated learning, which enables privacy-preserving multi-center collaboration without sharing raw patient data. Since DCA-MT is formulated as a general teacher–student mutual teaching framework operating on deep features and prediction logits, it can potentially be integrated with federated optimization by performing local mutual teaching on each client and aggregating model updates on a server. We will further investigate how to handle non-IID data across institutions, reduce communication overhead introduced by dual networks and attention modules via parameter-efficient sharing or distillation-to-lightweight models, and incorporate privacy-enhancing techniques such as secure aggregation or differential privacy. In addition, we will evaluate DCA-MT on broader datasets and more diverse backbones to further validate its generalization and scalability in real-world clinical environments.
