# Supplementary material for: Dual cross-attentive mutual teaching for semi-supervised 3D medical segmentation
Source: PLoS One. 2026 Jun 30;21(6):e0352358. doi: 10.1371/journal.pone.0352358 (PMC13318008; doi:10.1371/journal.pone.0352358)
Supplement: Attachment — Submitted filename: Response to Reviewers.docx [file pone.0352358.s002.docx]

**Response to Reviewers**

**Manuscript Title:** *Dual Cross-Attentive Mutual Teaching for Semi-Supervised 3D Medical Segmentation*

**Manuscript ID:**
**Corresponding Author:**

Dear Editor and Reviewers,

We sincerely thank you for your careful reading of our manuscript and for the constructive comments and suggestions. We have revised the paper thoroughly and believe the manuscript has been significantly improved in terms of motivation, novelty clarification, methodological presentation, experimental protocol transparency, computational analysis, and discussion of limitations and future directions.

**Response to Reviewer #1**

**Comment 1**

**Reviewer #1:** The motivation is not sufficiently clear, and the distinction between the proposed approach and recent dual-teacher or mutual-learning frameworks is not adequately discussed. The overall pipeline appears closely aligned with commonly used semi-supervised segmentation paradigms, making it difficult to identify a clear conceptual gap that this work aims to address.

**Response:**
Thank you for this insightful comment. We agree that the original manuscript did not sufficiently articulate the conceptual gap and the distinctions from recent dual-teacher / mutual-learning semi-supervised segmentation frameworks. In the revised version, we substantially strengthened the motivation and positioning of DCA-MT from both a conceptual and methodological perspective.

First, we explicitly clarify the conceptual gap our work targets: many existing dual-teacher or mutual-learning paradigms mainly rely on prediction-level consistency (e.g., EMA-based teacher guidance, pseudo-label agreement, or output-level co-training), while the cross-model interaction is often weakly coupled and coarse-grained, especially in 3D medical segmentation where semantic ambiguity and boundary sensitivity are prominent. This can lead to feature-space misalignment and unstable pseudo supervision when training with scarce labels and abundant unlabeled volumes. We now state this motivation more clearly and provide a more direct discussion of why prediction-level agreement alone may be insufficient for robust 3D anatomical delineation.

Second, we clarify how DCA-MT differs from prior dual-teacher/mutual-learning frameworks. Our method introduces an explicit semantic-level bidirectional interaction pathway via dual cross-attention at deep encoder features, enabling the teacher and student branches to *query and respond to each other* in high-level representations before decoding, rather than only aligning outputs. In addition, we emphasize that our bidirectional mutual distillation is designed to keep the teacher adaptive to patterns discovered from unlabeled data, mitigating the common issue of slow or stale EMA teacher updates. Finally, we highlight that DCA-MT couples this semantic interaction with high-dimensional feature alignment (MMD) and inter-/intra-class separability constraints, which jointly address global distribution mismatch and class confusion—an aspect not explicitly handled in many related pipelines.

**Comment 2**

Claims such as “for the first time” regarding the use of cross-attention between teacher and student networks are not well supported. In fact, numerous prior works have already explored similar cross-model or cross-branch attention mechanisms. These claims should either be substantiated with a careful comparison or rephrased to avoid overstatement.

**Response:**
Thank you for pointing this out. We fully agree that the phrase “for the first time” was not sufficiently justified and could be interpreted as an overstatement, given that cross-model/cross-branch attention mechanisms have been explored in prior literature. In the revised manuscript, we have removed all “for the first time” claims and rephrased the related statements to be accurate and appropriately scoped.

Importantly, our contribution is not claiming the first use of attention across models. Instead, we clarify that our novelty lies in the specific role and integration of cross-attention within the proposed semi-supervised 3D segmentation framework: (i) we employ a bidirectional cross-attention interaction at the deepest semantic encoding level to establish an explicit *query–response* pathway between teacher and student representations (beyond output-level agreement), and (ii) this semantic exchange is tightly coupled with bidirectional mutual distillation and feature-space alignment (MMD + inter-/intra-class constraints), forming a closed-loop collaborative learning process tailored to stabilizing pseudo supervision in 3D medical segmentation. We also expanded the related work discussion and added citations to better acknowledge prior cross-attention/mutual-learning style designs.

**Comment 3**

The mathematical formulations lack clarity in several places. Some variables and symbols are introduced without proper definition, which makes the loss design and optimization process difficult to follow.

**Response:**

Thank you for the constructive feedback. We agree that parts of the mathematical formulation in the original manuscript were not sufficiently clear, particularly regarding the introduction of symbols and the definition of variables used in the loss functions and optimization pipeline. In the revision, we have improved the presentation to make the formulation self-contained and easier to follow. Specifically, we (i) add explicit definitions for all symbols when they first appear (e.g., feature tensors, logits, pseudo-labels, index sets for same-/different-class pairs, kernel bandwidth, temperature, and balancing coefficients), (ii) unify notation across sections to avoid inconsistent symbols for the same concept, (iii) clarify tensor dimensions and where operations are applied (voxel-level vs. sample-level, encoder feature level vs. output logits), and (iv) provide a short step-by-step description of the training procedure indicating when each loss term is computed and how the overall objective is optimized. These clarifications improve readability and reproducibility without changing the underlying method.

**Comment 4**

The comparison baselines appear somewhat outdated. It would strengthen the empirical evaluation to include more recent and representative methods, such as “Evidence-based uncertainty-aware semi-supervised medical image segmentation”, to better demonstrate the competitiveness of the proposed approach.

**Response:**

Thank you for the suggestion. We agree that including more recent and representative baselines can further strengthen the empirical evaluation and better position DCA-MT against the latest advances. At the current revision stage, we focus on ensuring strict fairness and reproducibility for the selected baselines by implementing them under the same backbone and experimental protocol, and we have clarified this comparison protocol in the manuscript. Meanwhile, we acknowledge the reviewer’s recommendation and have added a dedicated discussion in the revised manuscript noting that recently proposed uncertainty-/evidence-based semi-supervised segmentation frameworks (including “Evidence-based uncertainty-aware semi-supervised medical image segmentation”) are highly relevant and represent an important direction for future comparison. We explicitly state that a more comprehensive benchmark that includes such recent methods will be conducted and reported in our extended experiments in future work (e.g., on additional datasets and multi-center settings), as these methods often involve non-trivial implementation details and training schedules that require careful integration to ensure a truly fair comparison.

**Comment 5**

The proposed framework appears largely decoupled from the specific backbone architecture. It is unclear whether and how the method generalizes to other segmentation networks beyond the chosen backbone. A discussion or experiment demonstrating backbone-agnostic applicability would improve the paper.
**Response:**

Thank you for this valuable suggestion. We agree that the original manuscript did not sufficiently demonstrate whether DCA-MT is backbone-agnostic. In the revised version, we clarify that DCA-MT is designed as a training framework operating on (i) deep encoder features and (ii) prediction logits, and thus can be integrated with a wide range of 3D encoder–decoder segmentation backbones with minimal modification. Concretely, the proposed modules—feature distribution alignment (MMD), inter-/intra-class separability constraints, bidirectional cross-attention, and bidirectional mutual distillation—do not rely on any VNet-specific operators. The only practical requirement is that the two branches expose a deep semantic feature map of matched resolution/channel dimension, which can be obtained from the deepest encoder stage of common backbones.

To substantiate this claim, we added an additional experiment using an alternative 3D segmentation backbone (nnU-Net) under the same semi-supervised protocol and labeled ratio. The results show that DCA-MT consistently improves performance over the corresponding baseline backbone, indicating that the proposed framework generalizes beyond VNet.

**Comment 6**

The experimental settings are insufficiently detailed. It is not clearly stated whether all compared semi-supervised methods adopt the same segmentation architecture. In addition, important details regarding training strategies, hyperparameter selection, and optimization protocols are missing and should be explicitly reported to ensure fair comparison and reproducibility.
**Response:**

Thank you for this helpful comment. We agree that the original manuscript did not provide sufficient implementation details and did not clearly state whether all compared methods share the same segmentation backbone, which could affect the perceived fairness and reproducibility. In the revised manuscript, we have substantially expanded the Experimental Settings to clearly specify the fair-comparison protocol, the training/optimization pipeline, and the hyperparameter selection strategy.

(1) Backbone consistency for fair comparison. We now explicitly state that, unless otherwise noted, all compared semi-supervised methods are implemented using the same 3D segmentation backbone (VNet) and the same data preprocessing and inference pipeline on each dataset. For methods with an official implementation or method-specific training schedules, we follow the original papers/official codes as closely as possible, and we clearly describe any necessary adaptations to the VNet backbone (and keep all other settings consistent) to ensure a fair comparison.

(2) Detailed training strategies and optimization protocols. We added missing details including: labeled/unlabeled batch composition, total training iterations, optimizer configuration, learning-rate schedule, EMA update rule for the teacher, data augmentations, patch sampling strategy, and sliding-window inference configuration. These additions make the training pipeline fully reproducible and ensure that baselines are trained under consistent settings.

(3) Hyperparameter selection and reproducibility. We now report all loss-weight coefficients (α, β, γ, λ₁, λ₂, etc.) and explain how they were determined (initialization from prior work followed by tuning on a held-out validation set with a grid search). We also include implementation details such as PyTorch version, GPU hardware, and random seed setting to facilitate reproducibility.

We believe these revisions address the reviewer’s concerns by making the experimental setup explicit, fair across methods, and reproducible.

**Comment 7**

The future work discussion could be expanded. In particular, it would be interesting to explore whether this framework can be extended to distributed or federated learning scenarios, as suggested by recent studies such as “Patient-level anatomy meets scanning-level physics: Personalized federated low-dose CT denoising empowered by large language model”. The authors are encouraged to add a dedicated discussion section on this direction.
**Response:**

Thank you for this insightful suggestion. We agree that the original manuscript’s future work discussion was brief and did not address the promising direction of distributed/federated learning (FL) for privacy-preserving medical applications. In the revised manuscript, we add a dedicated discussion subsection to explore how DCA-MT could be extended to federated and distributed settings, and we cite representative recent studies (including the paper suggested by the reviewer). Specifically, we discuss (i) how the teacher–student mutual teaching paradigm can be implemented under federated optimization, (ii) how to handle client heterogeneity and non-IID data via local adaptation and uncertainty-aware aggregation, and (iii) practical considerations such as communication overhead of dual networks and potential strategies (e.g., compressing teacher updates, distilling to a lightweight global student, or sharing only. We believe this addition better positions our work for real-world clinical deployment where data sharing is restricted by privacy regulations.

**Comment 8**

The computational cost of the proposed method is not discussed. Given the use of dual networks, cross-attention modules, and multiple loss terms, an analysis of training and inference complexity would be valuable.
**Response:**

Thank you for this important comment. We agree that the original manuscript did not include a dedicated analysis of computational cost, which is particularly relevant given the dual-branch design, cross-attention module, and multi-term objectives. In the revised manuscript, we add a Computational Cost subsection to report both theoretical complexity and empirical runtime/memory statistics.

Specifically, we (i) clarify that the proposed cross-attention is inserted at the deepest encoder stage where the spatial resolution is smallest, which keeps its additional complexity bounded; (ii) discuss that MMD and inter-/intra-class constraints are computed on deep features and introduce negligible overhead compared with 3D convolutional forward/backward passes; and (iii) report measured training time per iteration, GPU memory footprint, and inference time per volume under the same hardware and input configuration. We also clarify the inference strategy: the final prediction is obtained by averaging the two branches, and no extra optimization steps are required at test time beyond a standard forward pass. These additions allow readers to understand the computational trade-offs and facilitate practical deployment decisions.

**Comment 9**

The discussion of limitations is relatively brief. A more thorough analysis of potential failure modes, scalability issues, or scenarios where the method may underperform would improve the overall transparency and completeness of the manuscript.
**Response:**

Thank you for the valuable suggestion. We agree that the original manuscript provided only a brief limitations discussion. In the revised version, we have expanded the discussion to improve transparency by explicitly analyzing potential failure modes, scalability concerns, and scenarios where DCA-MT may underperform. Concretely, we discuss: (i) sensitivity to pseudo-label noise under severe domain shift or extremely low labeled ratio, which may propagate errors through mutual distillation; (ii) the risk of reduced benefit when the two branches become overly similar (loss of diversity) or when attention exchanges noisy semantics at early training stages; (iii) limitations in cases with highly ambiguous boundaries, very small target structures, or heavy artifacts/low SNR where cross-attention may amplify spurious features; and (iv) scalability and efficiency issues due to the dual-branch design (memory/compute), especially for large-volume inference and deployment. We also outline practical mitigation strategies such as confidence/uncertainty-based pseudo-label filtering, warm-up scheduling for cross-attention and bidirectional distillation, lightweight distillation for deployment, and extending to more diverse backbones and multi-center data.

**Response to Reviewer #2**

**Comment 1**

The proposed framework integrates several well-established components, including a teacher–student paradigm, mutual learning, maximum mean discrepancy (MMD) loss, contrastive constraints, and cross-attention mechanisms, all of which have been extensively explored in prior semi-supervised medical image segmentation studies. While the combination appears effective, the manuscript does not clearly articulate what fundamentally differentiates DCA-MT from existing mutual-teaching or cross-attention-based semi-supervised frameworks. The authors are encouraged to explicitly clarify the conceptual novelty and provide a more precise comparison with closely related methods.
Mutual teaching and bidirectional knowledge distillation are increasingly common in recent literature. The manuscript would benefit from a clearer explanation of how the proposed bidirectional distillation differs from prior mutual learning or co-training approaches. For example, does the crossed attention mechanism fundamentally alter the knowledge transfer pathway, or is it primarily an architectural enhancement?
Some advanced papers related semi-supervised medical image segmentation are suggested to review and cite.

**Response:**

Thank you for the insightful and constructive comments. We agree that the individual components involved in our framework (teacher–student learning, mutual learning, MMD-based alignment, contrastive constraints, and attention mechanisms) have been investigated in prior semi-supervised segmentation studies, and we acknowledge that the original manuscript did not sufficiently articulate what fundamentally differentiates DCA-MT from existing mutual-teaching or cross-attention-based frameworks. In the revised manuscript, we therefore strengthen the conceptual motivation and explicitly clarify the key novelty of DCA-MT as a *closed-loop mutual teaching framework* with an *explicit semantic interaction pathway* in the deep feature space. Specifically, unlike many mutual-learning or co-training approaches that primarily couple models through prediction-level consistency or pseudo-label agreement (often resulting in one-way or loosely coupled supervision), DCA-MT introduces a dual cross-attention “semantic bridge” at the highest-level encoder representations, where the teacher and student features can query each other (T→S and S→T) before decoding. This design does not serve as a merely architectural embellishment; rather, it changes the knowledge transfer pathway by enabling targeted, attention-weighted feature exchange at the semantic level, which in turn stabilizes and strengthens the subsequent two-way distillation. As a result, the teacher is not only an EMA-stabilized supervisor, but can also be adaptively refined through student feedback learned from unlabeled data, alleviating outdated teacher priors and improving adaptation to unlabeled distribution shift. In addition, beyond global distribution alignment via MMD, we incorporate class-structured inter-/intra-class constraints to explicitly enhance feature separability in the aligned space, mitigating foreground–background confusion that may remain even when global alignment is achieved. To better position our work, we also revise the Related Work section to include and discuss the recommended literature, especially the prototype-based semi-supervised medical segmentation and cross-sample consistency learning (e.g., MICCAI 2023 work), and we additionally mention the suggested cross-domain advances (contrastive learning, multigranularity learning, and diffusion-based distribution modeling) as methodological inspirations that highlight the broader relevance of structured representation optimization under limited supervision. Overall, these revisions clarify the conceptual novelty of DCA-MT, distinguish it more explicitly from closely related mutual-teaching paradigms, and improve the completeness and transparency of the manuscript’s positioning.
