# Supplementary material for: Dual cross-attentive mutual teaching for semi-supervised 3D medical segmentation
Source: PLoS One. 2026 Jun 30;21(6):e0352358. doi: 10.1371/journal.pone.0352358 (PMC13318008; doi:10.1371/journal.pone.0352358)
Supplement: Attachment — Submitted filename: Response_to_Reviewers_auresp_2.docx [file pone.0352358.s003.docx]

**Response to Reviewers**

**Manuscript ID:** PONE-D-25-64533R1

**Title:** Dual Cross-Attentive Mutual Teaching for Semi-Supervised 3D Medical Segmentation

Dear Academic Editor and Reviewers,

We sincerely thank the Academic Editor and both Reviewers for their constructive and insightful comments. We have carefully revised the manuscript and addressed all concerns raised during the review process. We believe these revisions have substantially improved the quality and clarity of the manuscript. Below, we provide a point-by-point response to each comment. All corresponding revisions have been highlighted in the revised manuscript.

# Summary of Revisions

1. Added comparison with EVIL-3D in the main benchmark table and updated Table 3.

2. Added a computational cost table reporting parameters, GMACs, training time, and inference time.

3. Expanded the discussion of efficiency and practical deployment trade-offs.

These revisions appear primarily in the sections “Comparison with Other Methods” and “Computational Cost and Efficiency”, as well as in the updated discussion and conclusion.

# Response to Reviewer #1

**Comment:**

*“The authors have addressed most of my concerns. However, the compared methods are kind of outdated, the authors should compare more advanced methods to illustrated the effectiveness as I mentioned in the first round review. Please compare the proposed method with the works mentioned in the first round and other methods.”*

**Response:**

We thank the Reviewer for this valuable suggestion. We agree that comparisons with more recent and competitive semi-supervised segmentation methods are important to better demonstrate the effectiveness of our proposed method. In the revised manuscript, we expanded the comparison by including the advanced methods mentioned in the first-round review wherever a fair and meaningful comparison was feasible. In particular, we added EVIL (Chen et al., Computers in Biology and Medicine, 2024) as an additional advanced baseline. Since the original EVIL framework was designed for 2D segmentation tasks, we implemented a 3D VNet-based adaptation (denoted as EVIL-3D) under the same training and evaluation protocol as our method to ensure a fair comparison on the LA dataset. Specifically, the evidential branch was adapted to produce Dirichlet-based outputs on top of a 3D VNet, and the uncertainty masking strategy was retained following the original design. This allows EVIL-3D to be evaluated fairly under the same 10% labeled setting used in our experiments. We also carefully considered the other methods mentioned in the first-round review. However, some of them are not directly comparable to our task setting: DHC is tailored to class-imbalanced multi-organ CT segmentation; SCP is a 2D semi-supervised method evaluated on 2D datasets; and DeSCO depends on orthogonal annotation settings rather than the standard full-mask semi-supervised protocol used in our study. We therefore clarified these points in the revised manuscript instead of including potentially unfair quantitative comparisons.

The updated comparison results on the LA dataset are shown below.

| Method | Venue | Dice (%) | Jaccard (%) | 95HD (mm) | ASD (mm) |
| --- | --- | --- | --- | --- | --- |
| VNet | - | 72.38 | 58.26 | 19.35 | 5.89 |
| UA-MT | NeurIPS 2017 | 76.10 | 62.62 | 10.84 | 2.43 |
| SASSNet | MICCAI 2020 | 77.66 | 64.08 | 10.93 | 3.05 |
| DTC | AAAI 2021 | 78.27 | 64.75 | 8.36 | 2.25 |
| BCP | CVPR 2023 | 82.91 | 70.97 | 6.43 | 2.25 |
| MCF | CVPR 2023 | 75.00 | 61.27 | 11.59 | 3.27 |
| GA | ECCV 2024 | 83.02 | 71.04 | 6.89 | 2.02 |
| EVIL-3D | CBM 2024 | 88.10 | 78.98 | 13.74 | 3.21 |
| DCA-MT (Ours) | - | 83.65 | 72.59 | 3.21 | 1.37 |

Although EVIL-3D achieves a higher Dice score than DCA-MT, its boundary-based metrics remain substantially worse. Specifically, EVIL-3D obtains 95HD = 13.74 mm and ASD = 3.21 mm, whereas DCA-MT achieves 95HD = 3.21 mm and ASD = 1.37 mm. This indicates that while EVIL-3D improves volumetric overlap, it is less effective in capturing precise anatomical boundaries. In contrast, the proposed DCA-MT explicitly performs bidirectional feature interaction between the teacher and student branches through cross-attention, which encourages more accurate structural alignment and suppresses spurious boundary predictions. As a result, our method shows clear advantages in boundary precision, which is particularly important for downstream clinical applications such as treatment planning and surgical guidance. These results demonstrate that the superiority of DCA-MT, especially in terms of surface-distance metrics, is not due to comparisons with outdated baselines, but remains evident even when evaluated against stronger and more recent methods. We have incorporated these additional experiments and the corresponding discussion into the revised manuscript in the section “Comparison with Other Methods” and updated Table 3 accordingly.

# Response to Reviewer #2

**Comment:**

*“It is necessary to take into account the network training time, the number of model parameters, and the inference time per case. These metrics should be reported, compared with other methods, and further analyzed.”*

**Response:**

We thank the Reviewer for this important suggestion. We fully agree that computational efficiency is an essential aspect for evaluating the practical applicability of a segmentation method. In response, we expanded the manuscript by adding a dedicated comparison of model complexity and runtime efficiency. Specifically, in the revised manuscript, we added a new table reporting the number of parameters, GMACs, training time per iteration, and inference time per case for all compared methods under the same hardware and software environment (NVIDIA GeForce RTX 3090, PyTorch 2.4.0, FP32) to ensure fairness.

| **Method** | **Params (M)** | **GMACs/patch** | **Train (ms/iter)** | **Infer-LA (s/vol)** | **Infer-PA (s/vol)** |
| --- | --- | --- | --- | --- | --- |
| VNet (backbone) | 9.44 | 40.27 | 58.8 | 2.63 | 0.31 |
| UA-MT | 9.44† | 40.27† | 132.6 | 2.74† | 0.35† |
| SASSNet | 9.44† | 40.27† | 132.9 | 2.74† | 0.35† |
| DTC | 9.44† | 40.27† | 133.1 | 2.74† | 0.35† |
| BCP | 9.44† | 40.27† | 134.1 | 2.74† | 0.35† |
| MCF | 18.89 | 80.55 | 267.2 | 4.49 | 0.69 |
| GA | 9.44† | 40.27† | 134.0 | 2.74† | 0.35† |
| **DCA-MT (Ours)** | **19.15** | **80.55** | **177.2** | **4.55** | **0.66** |

† UA-MT, SASSNet, DTC, BCP, and GA adopt a mean-teacher framework with two branches during training but only a single branch during inference; therefore, their inference cost is equivalent to that of a single VNet.

Several observations can be made from the new results. First, the parameter overhead introduced by DCA-MT is limited. The total parameter count is 19.15M, which is only slightly larger than that of other dual-branch methods. The additional parameters mainly come from the proposed bidirectional cross-attention module, whose size is relatively small compared with the two VNet backbones. Second, the training cost of DCA-MT is moderate. Its training time is 177.2 ms per iteration, which is higher than conventional mean-teacher-based methods but still substantially lower than MCF. This additional cost comes from the cross-attention interaction and feature-level consistency constraints, but remains manageable in practice. Third, the inference time of DCA-MT is mainly determined by its dual-branch inference design. Since both branches are used during prediction and their outputs are fused, the inference time is approximately 1.7 times that of single-branch mean-teacher methods. Nevertheless, this computational overhead is accompanied by a clear gain in segmentation quality, especially in boundary accuracy. For example, on the LA dataset, DCA-MT reduces 95HD from 6.43 mm (BCP) to 3.21 mm, indicating a much more precise surface delineation. Overall, these results show that DCA-MT achieves a favorable trade-off between segmentation accuracy and computational efficiency. We have added the above table and discussion to the revised manuscript in the subsection “Computational Cost and Efficiency”.

We sincerely hope that the revised manuscript satisfactorily addresses all concerns raised by the Reviewers and the Editor. We greatly appreciate the time and effort invested in evaluating our work.

**Data Availability Statement**:All data underlying the study are available at Figshare

(Codes:https://doi.org/10.6084/m9.figshare.32095765

Figures:https://doi.org/10.6084/m9.figshare.32095753)
