# Supplementary material for: Dual cross-attentive mutual teaching for semi-supervised 3D medical segmentation
Source: PLoS One. 2026 Jun 30;21(6):e0352358. doi: 10.1371/journal.pone.0352358 (PMC13318008; doi:10.1371/journal.pone.0352358)
Supplement: Peer review history

# Peer review: Decision Letter 0

**DOI:** 10.1371/journal.pone.0352358.r001

**Authors:** Qian Zhang

30 Dec 2025

PONE-D-25-64533

Dual Cross-Attentive Mutual Teaching for Semi-Supervised 3D Medical Segmentation

PLOS One

Dear Dr. MA,

Thank you for submitting your manuscript to PLOS ONE. After careful consideration, we feel that it has merit but does not fully meet PLOS ONE’s publication criteria as it currently stands. Therefore, we invite you to submit a revised version of the manuscript that addresses the points raised during the review process.

Please submit your revised manuscript by Feb 13 2026 11:59PM. If you will need more time than this to complete your revisions, please reply to this message or contact the journal office at plosone@plos.org. When you're ready to submit your revision, log on to https://www.editorialmanager.com/pone/ and select the 'Submissions Needing Revision' folder to locate your manuscript file.

Please include the following items when submitting your revised manuscript:

- A letter that responds to each point raised by the academic editor and reviewer(s). You should upload this letter as a separate file labeled 'Response to Reviewers'.
- A marked-up copy of your manuscript that highlights changes made to the original version. You should upload this as a separate file labeled 'Revised Manuscript with Track Changes'.
- An unmarked version of your revised paper without tracked changes. You should upload this as a separate file labeled 'Manuscript'.

If you would like to make changes to your financial disclosure, please include your updated statement in your cover letter. Guidelines for resubmitting your figure files are available below the reviewer comments at the end of this letter.

If applicable, we recommend that you deposit your laboratory protocols in protocols.io to enhance the reproducibility of your results. Protocols.io assigns your protocol its own identifier (DOI) so that it can be cited independently in the future. For instructions see: https://journals.plos.org/plosone/s/submission-guidelines#loc-laboratory-protocols. Additionally, PLOS ONE offers an option for publishing peer-reviewed Lab Protocol articles, which describe protocols hosted on protocols.io. Read more information on sharing protocols at https://plos.org/protocols?utm_medium=editorial-email&utm_source=authorletters&utm_campaign=protocols.

We look forward to receiving your revised manuscript.

Kind regards,

Qian Zhang, Ph.D

Academic Editor

PLOS One

Journal Requirements:

When submitting your revision, we need you to address these additional requirements.

1. Please ensure that your manuscript meets PLOS ONE's style requirements, including those for file naming. The PLOS ONE style templates can be found at

https://journals.plos.org/plosone/s/file?id=wjVg/PLOSOne_formatting_sample_main_body.pdf and

https://journals.plos.org/plosone/s/file?id=ba62/PLOSOne_formatting_sample_title_authors_affiliations.pdf

2. Please note that PLOS One has specific guidelines on code sharing for submissions in which author-generated code underpins the findings in the manuscript. In these cases, we expect all author-generated code to be made available without restrictions upon publication of the work. Please review our guidelines at https://journals.plos.org/plosone/s/materials-and-software-sharing#loc-sharing-code and ensure that your code is shared in a way that follows best practice and facilitates reproducibility and reuse.

3. We note that you have indicated that there are restrictions to data sharing for this study. PLOS only allows data to be available upon request if there are legal or ethical restrictions on sharing data publicly. For more information on unacceptable data access restrictions, please see http://journals.plos.org/plosone/s/data-availability#loc-unacceptable-data-access-restrictions.

Before we proceed with your manuscript, please address the following prompts:

a) If there are ethical or legal restrictions on sharing a de-identified data set, please explain them in detail (e.g., data contain potentially identifying or sensitive patient information, data are owned by a third-party organization, etc.) and who has imposed them (e.g., a Research Ethics Committee or Institutional Review Board, etc.). Please also provide contact information for a data access committee, ethics committee, or other institutional body to which data requests may be sent.

b) If there are no restrictions, please upload the minimal anonymized data set necessary to replicate your study findings to a stable, public repository and provide us with the relevant URLs, DOIs, or accession numbers. For a list of recommended repositories, please see

https://journals.plos.org/plosone/s/recommended-repositories. You also have the option of uploading the data as Supporting Information files, but we would recommend depositing data directly to a data repository if possible.

We will update your Data Availability statement on your behalf to reflect the information you provide.

4. If the reviewer comments include a recommendation to cite specific previously published works, please review and evaluate these publications to determine whether they are relevant and should be cited. There is no requirement to cite these works unless the editor has indicated otherwise.

[Note: HTML markup is below. Please do not edit.]

Reviewers' comments:

Reviewer's Responses to Questions

**Comments to the Author**

1. Is the manuscript technically sound, and do the data support the conclusions?

The manuscript must describe a technically sound piece of scientific research with data that supports the conclusions. Experiments must have been conducted rigorously, with appropriate controls, replication, and sample sizes. The conclusions must be drawn appropriately based on the data presented.

Reviewer #1: Yes

Reviewer #2: Yes

**********

2. Has the statistical analysis been performed appropriately and rigorously?

Reviewer #1: Yes

Reviewer #2: Yes

**********

3. Have the authors made all data underlying the findings in their manuscript fully available?

The PLOS Data policy requires authors to make all data underlying the findings described in their manuscript fully available without restriction, with rare exception (please refer to the Data Availability Statement in the manuscript PDF file). The data should be provided as part of the manuscript or its supporting information, or deposited to a public repository. For example, in addition to summary statistics, the data points behind means, medians and variance measures should be available. If there are restrictions on publicly sharing data—e.g. participant privacy or use of data from a third party—those must be specified.

Reviewer #1: Yes

Reviewer #2: Yes

**********

4. Is the manuscript presented in an intelligible fashion and written in standard English?

PLOS ONE does not copyedit accepted manuscripts, so the language in submitted articles must be clear, correct, and unambiguous. Any typographical or grammatical errors should be corrected at revision, so please note any specific errors here.

Reviewer #1: No

Reviewer #2: Yes

**********

5. Review Comments to the Author

Please use the space provided to explain your answers to the questions above. You may also include additional comments for the author, including concerns about dual publication, research ethics, or publication ethics. (Please upload your review as an attachment if it exceeds 20,000 characters)

Reviewer #1: The paper presents a comprehensive semi-supervised segmentation framework that combines feature alignment, cross-attention, and bidirectional distillation in a unified architecture. Overall, I think the idea is okay. However, this paper could be further improved, my suggestion is Major Revision. The main concerns are as follows:

1. The motivation is not sufficiently clear, and the distinction between the proposed approach and recent dual-teacher or mutual-learning frameworks is not adequately discussed. The overall pipeline appears closely aligned with commonly used semi-supervised segmentation paradigms, making it difficult to identify a clear conceptual gap that this work aims to address.

2. Claims such as “for the first time” regarding the use of cross-attention between teacher and student networks are not well supported. In fact, numerous prior works have already explored similar cross-model or cross-branch attention mechanisms. These claims should either be substantiated with a careful comparison or rephrased to avoid overstatement.

3. The mathematical formulations lack clarity in several places. Some variables and symbols are introduced without proper definition, which makes the loss design and optimization process difficult to follow.

4. The comparison baselines appear somewhat outdated. It would strengthen the empirical evaluation to include more recent and representative methods, such as “Evidence-based uncertainty-aware semi-supervised medical image segmentation”, to better demonstrate the competitiveness of the proposed approach.

5. The proposed framework appears largely decoupled from the specific backbone architecture. It is unclear whether and how the method generalizes to other segmentation networks beyond the chosen backbone. A discussion or experiment demonstrating backbone-agnostic applicability would improve the paper.

6. The experimental settings are insufficiently detailed. It is not clearly stated whether all compared semi-supervised methods adopt the same segmentation architecture. In addition, important details regarding training strategies, hyperparameter selection, and optimization protocols are missing and should be explicitly reported to ensure fair comparison and reproducibility.

7. The future work discussion could be expanded. In particular, it would be interesting to explore whether this framework can be extended to distributed or federated learning scenarios, as suggested by recent studies such as “Patient-level anatomy meets scanning-level physics: Personalized federated low-dose CT denoising empowered by large language model”. The authors are encouraged to add a dedicated discussion section on this direction.

8. The computational cost of the proposed method is not discussed. Given the use of dual networks, cross-attention modules, and multiple loss terms, an analysis of training and inference complexity would be valuable.

9. The discussion of limitations is relatively brief. A more thorough analysis of potential failure modes, scalability issues, or scenarios where the method may underperform would improve the overall transparency and completeness of the manuscript.

Reviewer #2: The proposed framework integrates several well-established components, including a teacher–student paradigm, mutual learning, maximum mean discrepancy (MMD) loss, contrastive constraints, and cross-attention mechanisms, all of which have been extensively explored in prior semi-supervised medical image segmentation studies. While the combination appears effective, the manuscript does not clearly articulate what fundamentally differentiates DCA-MT from existing mutual-teaching or cross-attention-based semi-supervised frameworks. The authors are encouraged to explicitly clarify the conceptual novelty and provide a more precise comparison with closely related methods.

Mutual teaching and bidirectional knowledge distillation are increasingly common in recent literature. The manuscript would benefit from a clearer explanation of how the proposed bidirectional distillation differs from prior mutual learning or co-training approaches. For example, does the crossed attention mechanism fundamentally alter the knowledge transfer pathway, or is it primarily an architectural enhancement?

Some advanced papers related semi-supervised medical image segmentation are suggested to review and cite.

Z. Zhang et al., "A Time-frequency Aware Hierarchical Feature Optimization Method for SAR Jamming Recognition," in IEEE Transactions on Aerospace and Electronic Systems, doi: 10.1109/TAES.2025.3563141.

H. Tan et al., "Few-Shot SAR ATR via Multilevel Contrastive Learning and Dependence Matrix-Based Measurement," in IEEE Journal of Selected Topics in Applied Earth Observations and Remote Sensing, vol. 18, pp. 8175-8188, 2025, doi: 10.1109/JSTARS.2025.3549632

Zhang, Z. et al. (2023). Self-aware and Cross-Sample Prototypical Learning for Semi-supervised Medical Image Segmentation. In: Greenspan, H., et al. Medical Image Computing and Computer Assisted Intervention – MICCAI 2023. MICCAI 2023. Lecture Notes in Computer Science, vol 14221. Springer, Cham. https://doi.org/10.1007/978-3-031-43895-0_18

H.Zhou et al., "Deformation-Resilient Multigranularity Learning for Unaligned RGB–T Semantic Segmentation," in IEEE Transactions on Neural Networks and Learning Systems, doi: 10.1109/TNNLS.2025.3585105.

H. Tan et al., "Improved Distribution Difference Driven Diffusion Generative Method for AMOSR," in IEEE Transactions on Cognitive Communications and Networking, doi: 10.1109/TCCN.2025.3613534. keywords: {Calibration;Accuracy;Training;Frequency modulation;Diffusion models;Prototypes;Data mining

Li, Y., Shi, X., Tan, H. et al. Multi-representation domain attentive contrastive learning based unsupervised automatic modulation recognition. Nat Commun 16, 5951 (2025). https://doi.org/10.1038/s41467-025-60921-z

H. Zhou et al., "Exploring Self-image and Cross-image Consistency Learning for Remote Sensing Burned Area Segmentation," in IEEE Transactions on Circuits and Systems for Video Technology, doi: 10.1109/TCSVT.2025.3615718.

**********

6. PLOS authors have the option to publish the peer review history of their article (what does this mean?). If published, this will include your full peer review and any attached files.

If you choose “no”, your identity will remain anonymous but your review may still be made public.

**Do you want your identity to be public for this peer review?**  For information about this choice, including consent withdrawal, please see our Privacy Policy.

Reviewer #1: No

Reviewer #2: No

**********

[NOTE: If reviewer comments were submitted as an attachment file, they will be attached to this email and accessible via the submission site. Please log into your account, locate the manuscript record, and check for the action link "View Attachments". If this link does not appear, there are no attachment files.]

To ensure your figures meet our technical requirements, please review our figure guidelines: https://journals.plos.org/plosone/s/figures

You may also use PLOS’s free figure tool, NAAS, to help you prepare publication quality figures: https://journals.plos.org/plosone/s/figures#loc-tools-for-figure-preparation.

NAAS will assess whether your figures meet our technical requirements by comparing each figure against our figure specifications.

# Author comment: Author response to Decision Letter 1

**DOI:** 10.1371/journal.pone.0352358.r002

6 Feb 2026

Response to Reviewer #1

Comment 1

Reviewer #1: The motivation is not sufficiently clear, and the distinction between the proposed approach and recent dual-teacher or mutual-learning frameworks is not adequately discussed. The overall pipeline appears closely aligned with commonly used semi-supervised segmentation paradigms, making it difficult to identify a clear conceptual gap that this work aims to address.

Response:

Thank you for this insightful comment. We agree that the original manuscript did not sufficiently articulate the conceptual gap and the distinctions from recent dual-teacher / mutual-learning semi-supervised segmentation frameworks. In the revised version, we substantially strengthened the motivation and positioning of DCA-MT from both a conceptual and methodological perspective.

First, we explicitly clarify the conceptual gap our work targets: many existing dual-teacher or mutual-learning paradigms mainly rely on prediction-level consistency (e.g., EMA-based teacher guidance, pseudo-label agreement, or output-level co-training), while the cross-model interaction is often weakly coupled and coarse-grained, especially in 3D medical segmentation where semantic ambiguity and boundary sensitivity are prominent. This can lead to feature-space misalignment and unstable pseudo supervision when training with scarce labels and abundant unlabeled volumes. We now state this motivation more clearly and provide a more direct discussion of why prediction-level agreement alone may be insufficient for robust 3D anatomical delineation.

Second, we clarify how DCA-MT differs from prior dual-teacher/mutual-learning frameworks. Our method introduces an explicit semantic-level bidirectional interaction pathway via dual cross-attention at deep encoder features, enabling the teacher and student branches to query and respond to each other in high-level representations before decoding, rather than only aligning outputs. In addition, we emphasize that our bidirectional mutual distillation is designed to keep the teacher adaptive to patterns discovered from unlabeled data, mitigating the common issue of slow or stale EMA teacher updates. Finally, we highlight that DCA-MT couples this semantic interaction with high-dimensional feature alignment (MMD) and inter-/intra-class separability constraints, which jointly address global distribution mismatch and class confusion—an aspect not explicitly handled in many related pipelines.

Comment 2

Claims such as “for the first time” regarding the use of cross-attention between teacher and student networks are not well supported. In fact, numerous prior works have already explored similar cross-model or cross-branch attention mechanisms. These claims should either be substantiated with a careful comparison or rephrased to avoid overstatement.

Response:

Thank you for pointing this out. We fully agree that the phrase “for the first time” was not sufficiently justified and could be interpreted as an overstatement, given that cross-model/cross-branch attention mechanisms have been explored in prior literature. In the revised manuscript, we have removed all “for the first time” claims and rephrased the related statements to be accurate and appropriately scoped.

Importantly, our contribution is not claiming the first use of attention across models. Instead, we clarify that our novelty lies in the specific role and integration of cross-attention within the proposed semi-supervised 3D segmentation framework: (i) we employ a bidirectional cross-attention interaction at the deepest semantic encoding level to establish an explicit query–response pathway between teacher and student representations (beyond output-level agreement), and (ii) this semantic exchange is tightly coupled with bidirectional mutual distillation and feature-space alignment (MMD + inter-/intra-class constraints), forming a closed-loop collaborative learning process tailored to stabilizing pseudo supervision in 3D medical segmentation. We also expanded the related work discussion and added citations to better acknowledge prior cross-attention/mutual-learning style designs.

Comment 3

The mathematical formulations lack clarity in several places. Some variables and symbols are introduced without proper definition, which makes the loss design and optimization process difficult to follow.

Response:

Thank you for the constructive feedback. We agree that parts of the mathematical formulation in the original manuscript were not sufficiently clear, particularly regarding the introduction of symbols and the definition of variables used in the loss functions and optimization pipeline. In the revision, we have improved the presentation to make the formulation self-contained and easier to follow. Specifically, we (i) add explicit definitions for all symbols when they first appear (e.g., feature tensors, logits, pseudo-labels, index sets for same-/different-class pairs, kernel bandwidth, temperature, and balancing coefficients), (ii) unify notation across sections to avoid inconsistent symbols for the same concept, (iii) clarify tensor dimensions and where operations are applied (voxel-level vs. sample-level, encoder feature level vs. output logits), and (iv) provide a short step-by-step description of the training procedure indicating when each loss term is computed and how the overall objective is optimized. These clarifications improve readability and reproducibility without changing the underlying method.

Comment 4

The comparison baselines appear somewhat outdated. It would strengthen the empirical evaluation to include more recent and representative methods, such as “Evidence-based uncertainty-aware semi-supervised medical image segmentation”, to better demonstrate the competitiveness of the proposed approach.

Response:

Thank you for the suggestion. We agree that including more recent and representative baselines can further strengthen the empirical evaluation and better position DCA-MT against the latest advances. At the current revision stage, we focus on ensuring strict fairness and reproducibility for the selected baselines by implementing them under the same backbone and experimental protocol, and we have clarified this comparison protocol in the manuscript. Meanwhile, we acknowledge the reviewer’s recommendation and have added a dedicated discussion in the revised manuscript noting that recently proposed uncertainty-/evidence-based semi-supervised segmentation frameworks (including “Evidence-based uncertainty-aware semi-supervised medical image segmentation”) are highly relevant and represent an important direction for future comparison. We explicitly state that a more comprehensive benchmark that includes such recent methods will be conducted and reported in our extended experiments in future work (e.g., on additional datasets and multi-center settings), as these methods often involve non-trivial implementation details and training schedules that require careful integration to ensure a truly fair comparison.

Comment 5

The proposed framework appears largely decoupled from the specific backbone architecture. It is unclear whether and how the method generalizes to other segmentation networks beyond the chosen backbone. A discussion or experiment demonstrating backbone-agnostic applicability would improve the paper.

Response:

Thank you for this valuable suggestion. We agree that the original manuscript did not sufficiently demonstrate whether DCA-MT is backbone-agnostic. In the revised version, we clarify that DCA-MT is designed as a training framework operating on (i) deep encoder features and (ii) prediction logits, and thus can be integrated with a wide range of 3D encoder–decoder segmentation backbones with minimal modification. Concretely, the proposed modules—feature distribution alignment (MMD), inter-/intra-class separability constraints, bidirectional cross-attention, and bidirectional mutual distillation—do not rely on any VNet-specific operators. The only practical requirement is that the two branches expose a deep semantic feature map of matched resolution/channel dimension, which can be obtained from the deepest encoder stage of common backbones.

To substantiate this claim, we added an additional experiment using an alternative 3D segmentation backbone (nnU-Net) under the same semi-supervised protocol and labeled ratio. The results show that DCA-MT consistently improves performance over the corresponding baseline backbone, indicating that the proposed framework generalizes beyond VNet.

Comment 6

The experimental settings are insufficiently detailed. It is not clearly stated whether all compared semi-supervised methods adopt the same segmentation architecture. In addition, important details regarding training strategies, hyperparameter selection, and optimization protocols are missing and should be explicitly reported to ensure fair comparison and reproducibility.

Response:

Thank you for this helpful comment. We agree that the original manuscript did not provide sufficient implementation details and did not clearly state whether all compared methods share the same segmentation backbone, which could affect the perceived fairness and reproducibility. In the revised manuscript, we have substantially expanded the Experimental Settings to clearly specify the fair-comparison protocol, the training/optimization pipeline, and the hyperparameter selection strategy.

(1) Backbone consistency for fair comparison. We now explicitly state that, unless otherwise noted, all compared semi-supervised methods are implemented using the same 3D segmentation backbone (VNet) and the same data preprocessing and inference pipeline on each dataset. For methods with an official implementation or method-specific training schedules, we follow the original papers/official codes as closely as possible, and we clearly describe any necessary adaptations to the VNet backbone (and keep all other settings consistent) to ensure a fair comparison.

(2) Detailed training strategies and optimization protocols. We added missing details including: labeled/unlabeled batch composition, total training iterations, optimizer configuration, learning-rate schedule, EMA update rule for the teacher, data augmentations, patch sampling strategy, and sliding-window inference configuration. These additions make the training pipeline fully reproducible and ensure that baselines are trained under consistent settings.

(3) Hyperparameter selection and reproducibility. We now report all loss-weight coefficients (α, β, γ, λ₁, λ₂, etc.) and explain how they were determined (initialization from prior work followed by tuning on a held-out validation set with a grid search). We also include implementation details such as PyTorch version, GPU hardware, and random seed setting to facilitate reproducibility.

We believe these revisions address the reviewer’s concerns by making the experimental setup explicit, fair across methods, and reproducible.

Comment 7

The future work discussion could be expanded. In particular, it would be interesting to explore whether this framework can be extended to distributed or federated learning scenarios, as suggested by recent studies such as “Patient-level anatomy meets scanning-level physics: Personalized federated low-dose CT denoising empowered by large language model”. The authors are encouraged to add a dedicated discussion section on this direction.

Response:

Thank you for this insightful suggestion. We agree that the original manuscript’s future work discussion was brief and did not address the promising direction of distributed/federated learning (FL) for privacy-preserving medical applications. In the revised manuscript, we add a dedicated discussion subsection to explore how DCA-MT could be extended to federated and distributed settings, and we cite representative recent studies (including the paper suggested by the reviewer). Specifically, we discuss (i) how the teacher–student mutual teaching paradigm can be implemented under federated optimization, (ii) how to handle client heterogeneity and non-IID data via local adaptation and uncertainty-aware aggregation, and (iii) practical considerations such as communication overhead of dual networks and potential strategies (e.g., compressing teacher updates, distilling to a lightweight global student, or sharing only. We believe this addition better positions our work for real-world clinical deployment where data sharing is restricted by privacy regulations.

Comment 8

The computational cost of the proposed method is not discussed. Given the use of dual networks, cross-attention modules, and multiple loss terms, an analysis of training and inference complexity would be valuable.

Response:

Thank you for this important comment. We agree that the original manuscript did not include a dedicated analysis of computational cost, which is particularly relevant given the dual-branch design, cross-attention module, and multi-term objectives. In the revised manuscript, we add a Computational Cost subsection to report both theoretical complexity and empirical runtime/memory statistics.

Specifically, we (i) clarify that the proposed cross-attention is inserted at the deepest encoder stage where the spatial resolution is smallest, which keeps its additional complexity bounded; (ii) discuss that MMD and inter-/intra-class constraints are computed on deep features and introduce negligible overhead compared with 3D convolutional forward/backward passes; and (iii) report measured training time per iteration, GPU memory footprint, and inference time per volume under the same hardware and input configuration. We also clarify the inference strategy: the final prediction is obtained by averaging the two branches, and no extra optimization steps are required at test time beyond a standard forward pass. These additions allow readers to understand the computational trade-offs and facilitate practical deployment decisions.

Comment 9

The discussion of limitations is relatively brief. A more thorough analysis of potential failure modes, scalability issues, or scenarios where the method may underperform would improve the overall transparency and completeness of the manuscript.

Response:

Thank you for the valuable suggestion. We agree that the original manuscript provided only a brief limitations discussion. In the revised version, we have expanded the discussion to improve transparency by explicitly analyzing potential failure modes, scalability concerns, and scenarios where DCA-MT may underperform. Concretely, we discuss: (i) sensitivity to pseudo-label noise under severe domain shift or extremely low labeled ratio, which may propagate errors through mutual distillation; (ii) the risk of reduced benefit when the two branches become overly similar (loss of diversity) or when attention exchanges noisy semantics at early training stages; (iii) limitations in cases with highly ambiguous boundaries, very small target structures, or heavy artifacts/low SNR where cross-attention may amplify spurious features; and (iv) scalability and efficiency issues due to the dual-branch design (memory/compute), especially for large-volume inference and deployment. We also outline practical mitigation strategies such as confidence/uncertainty-based pseudo-label filtering, warm-up scheduling for cross-attention and bidirectional distillation, lightweight distillation for deployment, and extending to more diverse backbones and multi-center data.

Response to Reviewer #2

Comment 1

The proposed framework integrates several well-established components, including a teacher–student paradigm, mutual learning, maximum mean discrepancy (MMD) loss, contrastive constraints, and cross-attention mechanisms, all of which have been extensively explored in prior semi-supervised medical image segmentation studies. While the combination appears effective, the manuscript does not clearly articulate what fundamentally differentiates DCA-MT from existing mutual-teaching or cross-attention-based semi-supervised frameworks. The authors are encouraged to explicitly clarify the conceptual novelty and provide a more precise comparison with closely related methods.

Mutual teaching and bidirectional knowledge distillation are increasingly common in recent literature. The

Attachment Submitted filename: Response to Reviewers.docx

# Peer review: Decision Letter 1

**DOI:** 10.1371/journal.pone.0352358.r003

**Authors:** Qian Zhang

27 Mar 2026

PONE-D-25-64533R1

Dual Cross-Attentive Mutual Teaching for Semi-Supervised 3D Medical Segmentation

PLOS One

Dear Dr. MA,

Thank you for submitting your manuscript to PLOS ONE. After careful consideration, we feel that it has merit but does not fully meet PLOS ONE’s publication criteria as it currently stands. Therefore, we invite you to submit a revised version of the manuscript that addresses the points raised during the review process.

Please submit your revised manuscript by May 11 2026 11:59PM. If you will need more time than this to complete your revisions, please reply to this message or contact the journal office at plosone@plos.org. When you're ready to submit your revision, log on to https://www.editorialmanager.com/pone/ and select the 'Submissions Needing Revision' folder to locate your manuscript file.

Please include the following items when submitting your revised manuscript:

- A letter that responds to each point raised by the academic editor and reviewer(s). You should upload this letter as a separate file labeled 'Response to Reviewers'.
- A marked-up copy of your manuscript that highlights changes made to the original version. You should upload this as a separate file labeled 'Revised Manuscript with Track Changes'.
- An unmarked version of your revised paper without tracked changes. You should upload this as a separate file labeled 'Manuscript'.

If you would like to make changes to your financial disclosure, please include your updated statement in your cover letter. Guidelines for resubmitting your figure files are available below the reviewer comments at the end of this letter.

If applicable, we recommend that you deposit your laboratory protocols in protocols.io to enhance the reproducibility of your results. Protocols.io assigns your protocol its own identifier (DOI) so that it can be cited independently in the future. For instructions see: https://journals.plos.org/plosone/s/submission-guidelines#loc-laboratory-protocols. Additionally, PLOS ONE offers an option for publishing peer-reviewed Lab Protocol articles, which describe protocols hosted on protocols.io. Read more information on sharing protocols at https://plos.org/protocols?utm_medium=editorial-email&utm_source=authorletters&utm_campaign=protocols.

As the corresponding author, your ORCID iD is verified in the submission system and will appear in the published article. PLOS supports the use of ORCID, and we encourage all coauthors to register for an ORCID iD and use it as well. Please encourage your coauthors to verify their ORCID iD within the submission system before final acceptance, as unverified ORCID iDs will not appear in the published article. *Only*  the individual author can complete the verification step; PLOS staff *cannot*  verify ORCID iDs on behalf of authors.

We look forward to receiving your revised manuscript.

Kind regards,

Qian Zhang, Ph.D

Academic Editor

PLOS One

Journal Requirements:

If the reviewer comments include a recommendation to cite specific previously published works, please review and evaluate these publications to determine whether they are relevant and should be cited. There is no requirement to cite these works unless the editor has indicated otherwise.

Please review your reference list to ensure that it is complete and correct. If you have cited papers that have been retracted, please include the rationale for doing so in the manuscript text, or remove these references and replace them with relevant current references. Any changes to the reference list should be mentioned in the rebuttal letter that accompanies your revised manuscript. If you need to cite a retracted article, indicate the article’s retracted status in the References list and also include a citation and full reference for the retraction notice.

[Note: HTML markup is below. Please do not edit.]

Reviewers' comments:

Reviewer's Responses to Questions

**Comments to the Author**

1. If the authors have adequately addressed your comments raised in a previous round of review and you feel that this manuscript is now acceptable for publication, you may indicate that here to bypass the “Comments to the Author” section, enter your conflict of interest statement in the “Confidential to Editor” section, and submit your "Accept" recommendation.

Reviewer #1: (No Response)

Reviewer #2: (No Response)

**********

2. Is the manuscript technically sound, and do the data support the conclusions?

The manuscript must describe a technically sound piece of scientific research with data that supports the conclusions. Experiments must have been conducted rigorously, with appropriate controls, replication, and sample sizes. The conclusions must be drawn appropriately based on the data presented.

Reviewer #1: (No Response)

Reviewer #2: (No Response)

**********

3. Has the statistical analysis been performed appropriately and rigorously?

Reviewer #1: (No Response)

Reviewer #2: (No Response)

**********

4. Have the authors made all data underlying the findings in their manuscript fully available?

The PLOS Data policy requires authors to make all data underlying the findings described in their manuscript fully available without restriction, with rare exception (please refer to the Data Availability Statement in the manuscript PDF file). The data should be provided as part of the manuscript or its supporting information, or deposited to a public repository. For example, in addition to summary statistics, the data points behind means, medians and variance measures should be available. If there are restrictions on publicly sharing data—e.g. participant privacy or use of data from a third party—those must be specified.

Reviewer #1: (No Response)

Reviewer #2: (No Response)

**********

5. Is the manuscript presented in an intelligible fashion and written in standard English?

PLOS ONE does not copyedit accepted manuscripts, so the language in submitted articles must be clear, correct, and unambiguous. Any typographical or grammatical errors should be corrected at revision, so please note any specific errors here.

Reviewer #1: (No Response)

Reviewer #2: (No Response)

**********

6. Review Comments to the Author

Please use the space provided to explain your answers to the questions above. You may also include additional comments for the author, including concerns about dual publication, research ethics, or publication ethics. (Please upload your review as an attachment if it exceeds 20,000 characters)

Reviewer #1: The authors have addressed most of my concerns. However, the compared methods are kind of outdated, the authors should compare more advanced methods to illustrated the effectiveness as I mentioned in the first round review. Please compare the proposed method with the works mentioned in the first round and other methods.

Reviewer #2: It is necessary to take into account the network training time, the number of model parameters, and the inference time per case. These metrics should be reported, compared with other methods, and further analyzed.

**********

7. PLOS authors have the option to publish the peer review history of their article (what does this mean?). If published, this will include your full peer review and any attached files.

If you choose “no”, your identity will remain anonymous but your review may still be made public.

**Do you want your identity to be public for this peer review?**  For information about this choice, including consent withdrawal, please see our Privacy Policy.

Reviewer #1: No

Reviewer #2: No

**********

[NOTE: If reviewer comments were submitted as an attachment file, they will be attached to this email and accessible via the submission site. Please log into your account, locate the manuscript record, and check for the action link "View Attachments". If this link does not appear, there are no attachment files.]

To ensure your figures meet our technical requirements, please review our figure guidelines: https://journals.plos.org/plosone/s/figures

You may also use PLOS’s free figure tool, NAAS, to help you prepare publication quality figures: https://journals.plos.org/plosone/s/figures#loc-tools-for-figure-preparation.

NAAS will assess whether your figures meet our technical requirements by comparing each figure against our figure specifications.

# Author comment: Author response to Decision Letter 2

**DOI:** 10.1371/journal.pone.0352358.r004

1 May 2026

Response to Reviewers

Manuscript ID: PONE-D-25-64533R1

Title: Dual Cross-Attentive Mutual Teaching for Semi-Supervised 3D Medical Segmentation

Dear Academic Editor and Reviewers,

We sincerely thank the Academic Editor and both Reviewers for their constructive and insightful comments. We have carefully revised the manuscript and addressed all concerns raised during the review process. We believe these revisions have substantially improved the quality and clarity of the manuscript. Below, we provide a point-by-point response to each comment. All corresponding revisions have been highlighted in the revised manuscript.

Summary of Revisions

1. Added comparison with EVIL-3D in the main benchmark table and updated Table 3.

2. Added a computational cost table reporting parameters, GMACs, training time, and inference time.

3. Expanded the discussion of efficiency and practical deployment trade-offs.

These revisions appear primarily in the sections “Comparison with Other Methods” and “Computational Cost and Efficiency”, as well as in the updated discussion and conclusion.

Response to Reviewer #1

Comment:

“The authors have addressed most of my concerns. However, the compared methods are kind of outdated, the authors should compare more advanced methods to illustrated the effectiveness as I mentioned in the first round review. Please compare the proposed method with the works mentioned in the first round and other methods.”

Response:

We thank the Reviewer for this valuable suggestion. We agree that comparisons with more recent and competitive semi-supervised segmentation methods are important to better demonstrate the effectiveness of our proposed method. In the revised manuscript, we expanded the comparison by including the advanced methods mentioned in the first-round review wherever a fair and meaningful comparison was feasible. In particular, we added EVIL (Chen et al., Computers in Biology and Medicine, 2024) as an additional advanced baseline. Since the original EVIL framework was designed for 2D segmentation tasks, we implemented a 3D VNet-based adaptation (denoted as EVIL-3D) under the same training and evaluation protocol as our method to ensure a fair comparison on the LA dataset. Specifically, the evidential branch was adapted to produce Dirichlet-based outputs on top of a 3D VNet, and the uncertainty masking strategy was retained following the original design. This allows EVIL-3D to be evaluated fairly under the same 10% labeled setting used in our experiments. We also carefully considered the other methods mentioned in the first-round review. However, some of them are not directly comparable to our task setting: DHC is tailored to class-imbalanced multi-organ CT segmentation; SCP is a 2D semi-supervised method evaluated on 2D datasets; and DeSCO depends on orthogonal annotation settings rather than the standard full-mask semi-supervised protocol used in our study. We therefore clarified these points in the revised manuscript instead of including potentially unfair quantitative comparisons.

The updated comparison results on the LA dataset are shown below.

Method Venue Dice (%) Jaccard (%) 95HD (mm) ASD (mm)

VNet - 72.38 58.26 19.35 5.89

UA-MT NeurIPS 2017 76.10 62.62 10.84 2.43

SASSNet MICCAI 2020 77.66 64.08 10.93 3.05

DTC AAAI 2021 78.27 64.75 8.36 2.25

BCP CVPR 2023 82.91 70.97 6.43 2.25

MCF CVPR 2023 75.00 61.27 11.59 3.27

GA ECCV 2024 83.02 71.04 6.89 2.02

EVIL-3D CBM 2024 88.10 78.98 13.74 3.21

DCA-MT (Ours) - 83.65 72.59 3.21 1.37

Although EVIL-3D achieves a higher Dice score than DCA-MT, its boundary-based metrics remain substantially worse. Specifically, EVIL-3D obtains 95HD = 13.74 mm and ASD = 3.21 mm, whereas DCA-MT achieves 95HD = 3.21 mm and ASD = 1.37 mm. This indicates that while EVIL-3D improves volumetric overlap, it is less effective in capturing precise anatomical boundaries. In contrast, the proposed DCA-MT explicitly performs bidirectional feature interaction between the teacher and student branches through cross-attention, which encourages more accurate structural alignment and suppresses spurious boundary predictions. As a result, our method shows clear advantages in boundary precision, which is particularly important for downstream clinical applications such as treatment planning and surgical guidance. These results demonstrate that the superiority of DCA-MT, especially in terms of surface-distance metrics, is not due to comparisons with outdated baselines, but remains evident even when evaluated against stronger and more recent methods. We have incorporated these additional experiments and the corresponding discussion into the revised manuscript in the section “Comparison with Other Methods” and updated Table 3 accordingly.

Response to Reviewer #2

Comment:

“It is necessary to take into account the network training time, the number of model parameters, and the inference time per case. These metrics should be reported, compared with other methods, and further analyzed.”

Response:

We thank the Reviewer for this important suggestion. We fully agree that computational efficiency is an essential aspect for evaluating the practical applicability of a segmentation method. In response, we expanded the manuscript by adding a dedicated comparison of model complexity and runtime efficiency. Specifically, in the revised manuscript, we added a new table reporting the number of parameters, GMACs, training time per iteration, and inference time per case for all compared methods under the same hardware and software environment (NVIDIA GeForce RTX 3090, PyTorch 2.4.0, FP32) to ensure fairness.

Method Params (M) GMACs/patch Train (ms/iter) Infer-LA (s/vol) Infer-PA (s/vol)

VNet (backbone) 9.44 40.27 58.8 2.63 0.31

UA-MT 9.44† 40.27† 132.6 2.74† 0.35†

SASSNet 9.44† 40.27† 132.9 2.74† 0.35†

DTC 9.44† 40.27† 133.1 2.74† 0.35†

BCP 9.44† 40.27† 134.1 2.74† 0.35†

MCF 18.89 80.55 267.2 4.49 0.69

GA 9.44† 40.27† 134.0 2.74† 0.35†

DCA-MT (Ours) 19.15 80.55 177.2 4.55 0.66

† UA-MT, SASSNet, DTC, BCP, and GA adopt a mean-teacher framework with two branches during training but only a single branch during inference; therefore, their inference cost is equivalent to that of a single VNet.

Several observations can be made from the new results. First, the parameter overhead introduced by DCA-MT is limited. The total parameter count is 19.15M, which is only slightly larger than that of other dual-branch methods. The additional parameters mainly come from the proposed bidirectional cross-attention module, whose size is relatively small compared with the two VNet backbones. Second, the training cost of DCA-MT is moderate. Its training time is 177.2 ms per iteration, which is higher than conventional mean-teacher-based methods but still substantially lower than MCF. This additional cost comes from the cross-attention interaction and feature-level consistency constraints, but remains manageable in practice. Third, the inference time of DCA-MT is mainly determined by its dual-branch inference design. Since both branches are used during prediction and their outputs are fused, the inference time is approximately 1.7 times that of single-branch mean-teacher methods. Nevertheless, this computational overhead is accompanied by a clear gain in segmentation quality, especially in boundary accuracy. For example, on the LA dataset, DCA-MT reduces 95HD from 6.43 mm (BCP) to 3.21 mm, indicating a much more precise surface delineation. Overall, these results show that DCA-MT achieves a favorable trade-off between segmentation accuracy and computational efficiency. We have added the above table and discussion to the revised manuscript in the subsection “Computational Cost and Efficiency”.

We sincerely hope that the revised manuscript satisfactorily addresses all concerns raised by the Reviewers and the Editor. We greatly appreciate the time and effort invested in evaluating our work.

Attachment Submitted filename: Response_to_Reviewers_auresp_2.docx

# Peer review: Decision Letter 2

**DOI:** 10.1371/journal.pone.0352358.r005

**Authors:** Qian Zhang

9 Jun 2026

Dual Cross-Attentive Mutual Teaching for Semi-Supervised 3D Medical Segmentation

PONE-D-25-64533R2

Dear Dr. MA,

We’re pleased to inform you that your manuscript has been judged scientifically suitable for publication and will be formally accepted for publication once it meets all outstanding technical requirements.

Within one week, you’ll receive an e-mail detailing the required amendments. When these have been addressed, you’ll receive a formal acceptance letter and your manuscript will be scheduled for publication.

An invoice will be generated when your article is formally accepted. Please note, if your institution has a publishing partnership with PLOS and your article meets the relevant criteria, all or part of your publication costs will be covered. Please make sure your user information is up-to-date by logging into Editorial Manager at Editorial Manager® and clicking the ‘Update My Information' link at the top of the page. For questions related to billing, please contact billing support.

If your institution or institutions have a press office, please notify them about your upcoming paper to help maximize its impact. If they’ll be preparing press materials, please inform our press team as soon as possible -- no later than 48 hours after receiving the formal acceptance. Your manuscript will remain under strict press embargo until 2 pm Eastern Time on the date of publication. For more information, please contact onepress@plos.org.

Kind regards,

Qian Zhang, Ph.D

Academic Editor

PLOS One

Additional Editor Comments (optional):

Reviewers' comments:

Reviewer's Responses to Questions

**Comments to the Author**

1. If the authors have adequately addressed your comments raised in a previous round of review and you feel that this manuscript is now acceptable for publication, you may indicate that here to bypass the “Comments to the Author” section, enter your conflict of interest statement in the “Confidential to Editor” section, and submit your "Accept" recommendation.

Reviewer #1: All comments have been addressed

Reviewer #2: All comments have been addressed

**********

2. Is the manuscript technically sound, and do the data support the conclusions?

The manuscript must describe a technically sound piece of scientific research with data that supports the conclusions. Experiments must have been conducted rigorously, with appropriate controls, replication, and sample sizes. The conclusions must be drawn appropriately based on the data presented.

Reviewer #1: Yes

Reviewer #2: Yes

**********

3. Has the statistical analysis been performed appropriately and rigorously?

Reviewer #1: Yes

Reviewer #2: Yes

**********

4. Have the authors made all data underlying the findings in their manuscript fully available?

The PLOS Data policy requires authors to make all data underlying the findings described in their manuscript fully available without restriction, with rare exception (please refer to the Data Availability Statement in the manuscript PDF file). The data should be provided as part of the manuscript or its supporting information, or deposited to a public repository. For example, in addition to summary statistics, the data points behind means, medians and variance measures should be available. If there are restrictions on publicly sharing data—e.g. participant privacy or use of data from a third party—those must be specified.

Reviewer #1: Yes

Reviewer #2: Yes

**********

5. Is the manuscript presented in an intelligible fashion and written in standard English?

PLOS ONE does not copyedit accepted manuscripts, so the language in submitted articles must be clear, correct, and unambiguous. Any typographical or grammatical errors should be corrected at revision, so please note any specific errors here.

Reviewer #1: (No Response)

Reviewer #2: Yes

**********

6. Review Comments to the Author

Please use the space provided to explain your answers to the questions above. You may also include additional comments for the author, including concerns about dual publication, research ethics, or publication ethics. (Please upload your review as an attachment if it exceeds 20,000 characters)

Reviewer #1: Thanks for the effort, and I believe that the authors have well addressed my concerns. Hence, my suggestion is Accept.

Reviewer #2: The authors have made effort to improve the paper quality. All my concerns have been addressed. An acceptance is suggested.

**********

7. PLOS authors have the option to publish the peer review history of their article (what does this mean?). If published, this will include your full peer review and any attached files.

If you choose “no”, your identity will remain anonymous but your review may still be made public.

**Do you want your identity to be public for this peer review?**  For information about this choice, including consent withdrawal, please see our Privacy Policy.

Reviewer #1: No

Reviewer #2: No

**********

# Editor report: Acceptance letter

**DOI:** 10.1371/journal.pone.0352358.r006

**Authors:** Qian Zhang

PONE-D-25-64533R2

PLOS One

Dear Dr. MA,

I'm pleased to inform you that your manuscript has been deemed suitable for publication in PLOS One. Congratulations! Your manuscript is now being handed over to our production team.

At this stage, our production department will prepare your paper for publication. This includes ensuring the following:

* All references, tables, and figures are properly cited

* All relevant supporting information is included in the manuscript submission,

* There are no issues that prevent the paper from being properly typeset

You will receive further instructions from the production team, including instructions on how to review your proof when it is ready. Please keep in mind that we are working through a large volume of accepted articles, so please give us a few days to review your paper and let you know the next and final steps.

Lastly, if your institution or institutions have a press office, please let them know about your upcoming paper now to help maximize its impact. If they'll be preparing press materials, please inform our press team within the next 48 hours. Your manuscript will remain under strict press embargo until 2 pm Eastern Time on the date of publication. For more information, please contact onepress@plos.org.

You will receive an invoice from PLOS for your publication fee after your manuscript has reached the completed accept phase. If you receive an email requesting payment before acceptance or for any other service, this may be a phishing scheme. Learn how to identify phishing emails and protect your accounts at https://explore.plos.org/phishing.

If we can help with anything else, please email us at customercare@plos.org.

Thank you for submitting your work to PLOS ONE and supporting open access.

Kind regards,

PLOS ONE Editorial Office Staff

on behalf of

Dr. Qian Zhang

Academic Editor

PLOS One
